# Identification of Signaling Pathways for Early Embryonic Lethality and Developmental Retardation in *Sephs1^−/−^* Mice

**DOI:** 10.3390/ijms222111647

**Published:** 2021-10-28

**Authors:** Jeyoung Bang, Minguk Han, Tack-Jin Yoo, Lu Qiao, Jisu Jung, Jiwoon Na, Bradley A. Carlson, Vadim N. Gladyshev, Dolph L. Hatfield, Jin-Hong Kim, Lark Kyun Kim, Byeong Jae Lee

**Affiliations:** 1 Interdisciplinary Program in Bioinformatics, College of Natural Sciences, Seoul National University, Seoul 08826, Korea; 880419@snu.ac.kr (J.B.); han3270@snu.ac.kr (M.H.); 2 School of Biological Sciences, College of Natural Sciences, Seoul National University, Seoul 08826, Korea; yootackjin@snu.ac.kr (T.-J.Y.); qiaolu@snu.ac.kr (L.Q.); tabris0520@snu.ac.kr (J.J.); naji0708@snu.ac.kr (J.N.); jinhkim@snu.ac.kr (J.-H.K.); 3 Mouse Cancer Genetics Program, Center for Cancer Research, National Cancer Institute, National Institutes of Health, Bethesda, MD 20892, USA; carlsonb@dc37a.nci.nih.gov (B.A.C.); hatfielddolph@gmail.com (D.L.H.); 4 Department of Medicine, Brigham and Women’s Hospital, Harvard Medical School, Boston, MA 02115, USA; vgladyshev@rics.bwh.harvard.edu; 5 Severance Biomedical Science Institute, Graduate School of Medical Science, Brain Korea 21 Project, Gangnam Severance Hospital, Yonsei University College of Medicine, Seoul 06230, Korea

**Keywords:** selenium, selenoprotein, SEPHS1, early embryogenesis, embryonic lethality, reactive oxygen species

## Abstract

Selenophosphate synthetase 1 (SEPHS1) plays an essential role in cell growth and survival. However, the underlying molecular mechanisms remain unclear. In the present study, the pathways regulated by SEPHS1 during gastrulation were determined by bioinformatical analyses and experimental verification using systemic knockout mice targeting *Sephs1*. We found that the coagulation system and retinoic acid signaling were most highly affected by SEPHS1 deficiency throughout gastrulation. Gene expression patterns of altered embryo morphogenesis and inhibition of Wnt signaling were predicted with high probability at E6.5. These predictions were verified by structural abnormalities in the dermal layer of *Sephs1^−/−^* embryos. At E7.5, organogenesis and activation of prolactin signaling were predicted to be affected by *Sephs1* knockout. Delay of head fold formation was observed in the *Sephs1^−/−^* embryos. At E8.5, gene expression associated with organ development and insulin-like growth hormone signaling that regulates organ growth during development was altered. Consistent with these observations, various morphological abnormalities of organs and axial rotation failure were observed. We also found that the gene sets related to redox homeostasis and apoptosis were gradually enriched in a time-dependent manner until E8.5. However, DNA damage and apoptosis markers were detected only when the *Sephs1^−/−^* embryos aged to E9.5. Our results suggest that SEPHS1 deficiency causes a gradual increase of oxidative stress which changes signaling pathways during gastrulation, and afterwards leads to apoptosis.

## 1. Introduction

Selenium (Se) is an essential trace element required in the diet of humans and other forms of life. An adequate amount of selenium is essential to good health. For example, Se is implicated in cancer prevention, antiviral response, boosting the immune system, male reproduction, and embryo development [1,2,3]. In addition to those beneficial effects of Se, it is notable that Se also affects the progression of pregnancy. Specifically, Se levels and the activity of blood glutathione peroxidase were lower than average in women who had experienced miscarriage, premature birth, or preeclampsia [4,5]. Moreover, pregnant women who had low serum Se levels showed a high incidence of spontaneous miscarriage [6].

Se is the only trace element to be specified in the genetic code, and selenocysteine (Sec) is the 21st amino acid, which is incorporated into protein in response to the UGA codon during translation [7]. Selenoproteins contain single or multiple Sec residues in their active sites and are known to carry out important roles, often with the beneficial effects of Se described above [1,2]. One of most common functions of selenoproteins is to protect cells and tissues from oxidative stress by serving as reactive oxygen species (ROS) scavengers [8,9]. During Sec synthesis, selenophosphate serves as the selenium donor. Selenophosphate synthetase (SEPHS) catalyzes the reaction of selenophosphate synthesis, in which inorganic selenium and ATP are used as substrates [10]. There are two types of SEPHSs in higher eukaryotes, SEPHS1 and 2 [11]. Both isotypes have ATP binding and catalytic domains, and there is a high amino acid sequence homology between them. One of the biggest differences between SEPHS1 and 2 is that SEPHS2 contains Sec in the catalytic domain, while SEPHS1 does not. Instead, SEPHS1 contains threonine at the position corresponding to Sec in SEPHS2 [12]. Another feature is that only SEPHS2 has the ability to synthesize selenophosphate [13]. According to data from the International Mouse Phenotyping Consortium, whole-gene deletion of *Sephs2* did not show embryonic lethality but showed abnormalities in heart morphology of the knockout fetuses [14]. SEPHS1, however, is required for cell survival and proliferation [15].

The most prominent role of SEPHS1 is that it participates in the regulation of cellular redox homeostasis [3]. In *Drosophila*, a P-element insertion mutation in *Sephs1* (*SelD*) led to embryonic lethality following the loss of imaginal disc formation [16]. Subsequently, it was demonstrated that the embryonic lethality in *Drosophila* is mediated by ROS-induced apoptosis [17]. An in vitro study using SL2 cells, an embryonic cell line of *Drosophila*, showed that SEPHS1 deficiency induced ROS accumulation which in turn led to the inhibition of cell proliferation and glutamine-dependent megamitochondria formation [18]. In addition to studies in *Drosophila*, systemic knockout mice targeting *Sephs1* showed embryonic lethality and complete resorption by E14.5 [19]. Knockdown of *Sephs1* in a mouse embryonic cancer cell line (F9) showed the inhibition of cell proliferation by increased levels of ROS, specifically hydrogen peroxide [19]. Deficiency of SEPHS1 in F9 cells also reversed cancer malignancy characteristics such as cell invasion and anchorage independence. The expression levels of glutaredoxin 1 (*Glrx1*) and glutathione-s-transferase O1 (*GstO1*), which are involved in redox homeostasis, were significantly decreased by SEPHS1-deficiency in F9 cells.

Oxidative stress causes numerous types of damage during embryonic development by altering cellular macromolecules such as lipids, proteins, and nucleic acids [20]. Consequently, an affected embryo undergoes growth inhibition, development retardation, metabolic dysfunction, and apoptosis [21]. In the case of embryos cultured in vitro, high ROS levels have detrimental effects on embryo growth, but the addition of free radical scavengers recovers cells from the detrimental effects of ROS [22]. The importance of ROS during development was also demonstrated by regulating the expression of antioxidant genes. For example, disruption of thioredoxin 1 (*Txn1*) resulted in embryo hatching failure and lethality shortly after implantation [23]. Thioredoxin 2 (*Txn2*) mutation inhibited neural tube formation and induced massive apoptosis at E10.5 [24].

Although it is known that SEPHS1 plays an essential role for cell survival and proliferation, the underlying molecular and biochemical mechanisms have not been fully clarified. To elucidate the role of SEPHS1 during early embryogenesis, we generated systemic knockout mice targeting *Sephs1*. Pathways and genes regulated by SEPHS1 were predicted using various bioinformatical tools described herein, and the predicted pathways were verified by analyzing the anatomical structure of the developing embryo.

## 2. Results

### 2.1. RNA-seq Data Analysis

**Figure 1:**
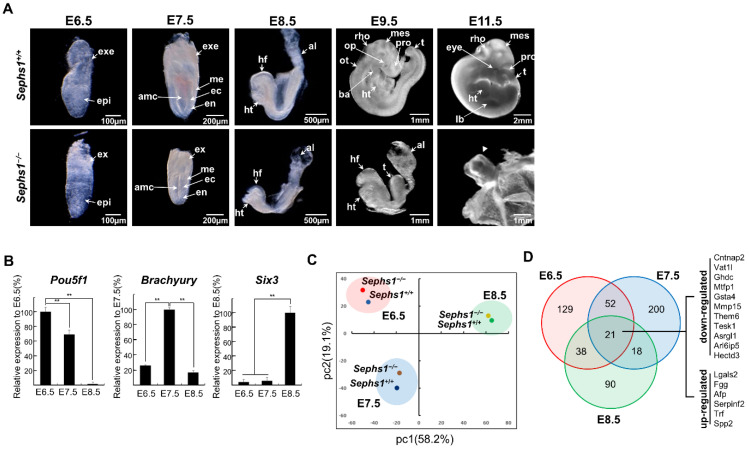
Transcriptome analysis of *Sephs1^−/−^* embryos. (**A**) Morphology of wild-type and *Sephs1^−/−^* embryos after dissection. The yolk sac was removed after E8.5. Arrowhead indicates absorbed *Sephs1^−/−^* embryo at E11.5 that attached to the yolk sac. (**B**) Real-time quantitative PCR of embryonic stage marker genes. Expression levels of *Pou5f1* (*Oct4*), *Brachyury* (*T*), and *Six3* were measured in wild-type embryos as described in Materials and Methods. ** indicates *p* < 0.01. (**C**) Principal component analysis of RNA-seq data. The percentages represent the variance captured by each principal components 1 and 2 in analysis. (**D**) Venn diagram of the number of DEGs overlapping between embryonic day E6.5-E8.5. DEG cut-off: max(FPKM) > 1, |Log2(Fold Change)| > 1 and *p* < 0.01. al, allantois; amc, amniotic cavity; ba, first branchial arch; ec, ectoderm; en, endoderm; epi, epiblast; exe, extra embryonic region; hf, head fold; ht, heart; lb, limb bud; me, mesoderm; mes, mesencephalon; op, optic vesicle; ot, otic vesicle; rho, rhombencephalon; t, tail; tel, telencephalon.

We previously reported that the systemic knockout targeting *Sephs1* in a mouse model resulted in the embryo beginning to show a difference in size at E7.5 and lethality at E11.5 [19]. In this study, the effect of SEPHS1 deficiency on early embryogenesis was analyzed in more detail during this period. Figure 1A shows whole-embryo images obtained by optical microscopy. There are no differences between wild-type (*Sephs1^+/+^* and *Sephs1^+/−^*) and the *Sephs1^−/−^* embryo at E6.5. The *Sephs1^−/−^* embryo began to show size differences from wild-type at E7.5, and the difference extended at E8.5, wherein head folds were observed to be less developed than in wild-type. In addition, the *Sephs1^−/−^* embryo does not turn in the final fetal position at E9.5 and is smaller than wild-type. Unlike in the wild-type embryo, optic and otic vesicles were not observed, although the allantois still remained, and three primary brain vesicles (prosencephalon, mesencephalon, and rhombencephalon) were observed as being immature. At E9.5, no vitelline vessel was found in the yolk sac (arrow in Figure S1A). At E10.5, the size of the *Sephs1^−/−^* embryo was dramatically reduced from that at E9.5 (data not shown) and the embryo appeared to be fully absorbed at E11.5 (arrowhead in panel E11.5 of Figure 1A). We used 32, 24, 34, 20, 17, and 10 embryos at E6.5, E7.5, E8.5, E9.5, E10.5, and E11.5, respectively (See Materials and Methods for more detailed information). All the embryos that had the same genotype and were prepared at the same embryonic day showed similar phenotypes both in size and in morphology.

In order to assess the effect of SEPHS1 deficiency on embryonic development and the related signaling pathways responsible for phenotypic changes, RNA-seq was performed using purified RNA from wild-type and *Sephs1^−/−^* embryos at the E6.5, E7.5, and E8.5 stages.

As shown in Figure 1B, *Pou5f1* (*Oct4*) and *Brachyury* (*T*) were expressed most abundantly at E6.5 and at E7.5, respectively, and *Six3* was expressed only at E8.5 in wild-type embryo. These results indicate that the pooled RNAs isolated from embryos at the same embryonic day were highly homogeneous. In addition, principal component analysis (PCA) was performed to examine the relationship between the read sets. PCA revealed that the differences in developmental stages are much more distinct than genotypic differences between wild-type and *Sephs1* knockout (Figure 1C). Differentially expressed genes (DEGs) were obtained at E6.5, E7.5 and E8.5 with the log_2_(fold change) cut off of ±1 at *p* < 0.01 (Table S1). There are 21 genes commonly affected by SEPHS1 deficiency at all three stages (Figure 1D). The functions of the down-regulated genes include those relating to organogenesis (*Cntnap2*, *Gata4*, *Mmp15*, *Asrgl1*, and *Arl6ip5*) and cell survival (*Hectd3*) [25,26,27]. Interestingly, the up-regulated genes showed region-specificity; *Fgg*, *Afp*, *Trf*, and *Serpinf2* in the extraembryonic region and *Spp2* in the placenta. Notably, *Galectin 2* (*Lgals2*), the most up-regulated DEG, is reported to be an oxidative stress-responsive gene shown to be up-regulated under H_2_O_2_ treatment [28]. These results suggest that SEPHS1-deficiency causes developmental retardation and the induction of oxidative stress. 

### 2.2. Pathway Analysis of Differentially Expressed Genes

**Figure 2:**
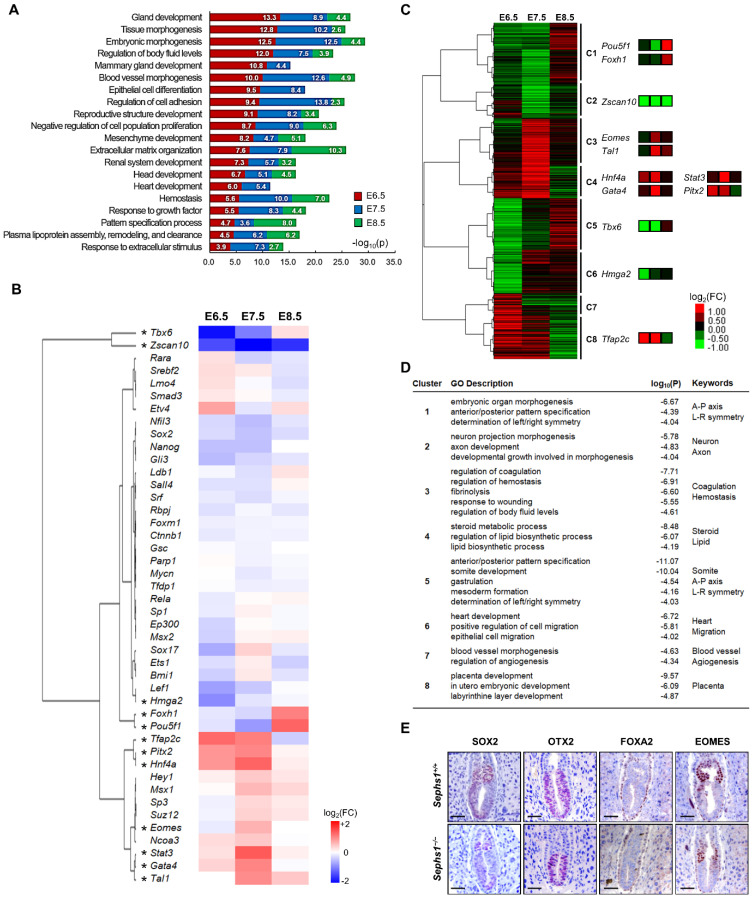
Pathway analysis of *Sephs1^−/−^* embryo and transcription factor prediction. (**A**) Metascape pathway enrichment analysis. Each gene ontology group was integrated into summary terms according to the significance. *p* < 0.01. (**B**) Transcription factors that regulate gastrulation-related DEGs in the *Sephs1^−/−^* embryo. Fold-change of transcription factor between wild-type and *Sephs1^−/−^* embryos was illustrated by heat map. * indicates DEGs. (**C**) Hierarchical clustering of DEGs according to their fold-change. The distance between DEGs was calculated via Pearson correlation, and clustered by the pairwise complete linkage method. Clusters were defined at the third branch from the base trunk. (**D**) GO analysis using clustered DEG sets. Cut off at *p* < 0.001. (**E**) Expression patterns of dermal layer marker genes at E6.5. Immunohistochemistry was performed with SOX2, OTX2, FOXA2, and EOMES as described in Materials and Methods. Scale bars represent 100 µm.

In order to identify the pathways that are regulated by SEPHS1, pathway enrichment analysis using Metascape was performed with DEGs (Figure 2A), with the log_2_(fold change) cut off of ± 0.5 at *p* < 0.01. At E6.5, embryo and tissue morphogenesis were significantly affected by *Sephs1* knockout. At E7.5 and E8.5, development of differentiated tissues such as ‘vascular morphogenesis’, ‘epithelial cell differentiation’, ‘mesenchymal development’, ‘head’ development’, and ‘heart development’ were greatly affected. Some of the predicted results in Figure 2A were consistent with the results of Ingenuity Pathway Analysis (IPA) (Table S2). For example, the genes included in ‘LXR/RXR activation’ predicted by IPA were also found in the ‘Regulation of body fluid level’ and ‘Plasma lipoprotein assembly, remodeling, and clearance’ predicted by the Metascape analysis. These genes participate in retinoic acid (RA) signaling. In addition, most of the genes in the ‘Coagulation system’ of IPA were included in the Metascape category of ‘Hemostasis’.

In order to identify the transcription factors targeting the DEGs during gastrulation, transcription factors that are known to be activated during gastrulation were selected from Transcriptional Regulatory Relationships Unraveled by Sentence-based Text mining (TRRUST) database. Among the gastrulation-specific transcription factors, the transcription factors that contain target genes (of which more than 50% are DEGs) were further selected to identify the transcription factors governing the expression of target DEGs (Figure 2B). Among the selected transcription factors, 12 transcription factors were DEG themselves (asterisks in Figure 2B). We defined these transcription factor genes as differentially expressed transcription factor genes (DTFGs). Interestingly, DTFGs were apt to be clustered together. The expression of DEGs by non-DTFGs, the transcription factors whose expression was not changed by *Sephs1* knockout, may be regulated indirectly by changes in protein stability, phosphorylation, and interaction with other co-regulators.

To analyze the expression pattern of DEGs, hierarchical clustering was performed (Figure 2C). Expression patterns of DTFGs (asterisks in Figure 2B) were indicated on the right of each cluster to which they belong. The DTFGs in the same cluster in Figure 2B were in the same or neighboring cluster in Figure 2C. For example, *Eomes*, *Tal1*, *Hnf4α*, *Gata4*, *Stat3*, and *Pitx2* were included in C3 and C4. This suggests that DTFGs are expressed in the same pattern with their target genes.

To identify biological processes that were most highly affected by SEPHS1 deficiency, gene ontology (GO) analysis was performed with DEGs belonging to each cluster (Figure 2D). As a result, genes in each cluster were found to regulate distinct pathways. For example, the genes in cluster 1 were predicted to regulate pathways participating in axis formation and the genes in cluster 2 in neuron development.

Since dermal layers of the embryos at the early gastrula stage determine the cell lineage leading to tissue differentiation, the gene expression pattern and/or morphological feature of each dermal layer are important to organ development at the following stages. We examined the expression levels and locations of dermal layer markers (*Sox2*, *Otx2*, *Foxa2*, and *Eomes*) selected from the transcription factors shown in Figure 2B.

Sex determining region Y-box 2 (*Sox2*) encodes a transcription factor that is essential for maintaining pluripotency of undifferentiated embryonic stem cells, but is also known to be expressed specifically in the ectoderm of both embryonic and extraembryonic regions during the gastrulation stage [29]. As shown in Figure 2E, the levels of *Sox2* expression were significantly decreased in the *Sephs1^−/−^* embryo compared to wild-type (log_2_(Fold Change) at E6.5 = −0.42). Orthodenticle homeobox 2 (*Otx2*), which encodes a member of the bicoid subfamily of homeodomain-containing transcription factors, is an embryonic mesoderm-specific gene that plays a key role in nervous system development [30]. The area in which *Otx2* was expressed was reduced to the bottom half of the *Sephs1^−/−^* embryo, while the size of the embryo was similar with that of wild-type. The fact that the structure of mesodermal layer was changed by SEPHS1-deficiency suggests that the development of connective tissues such as blood, blood vessels, muscles, and heart will proceed abnormally in the *Sephs1^−/−^* embryos, because these tissues are differentiated from the mesodermal lineage cells. Notably, the expression level of *Otx2* was not changed by *Sephs1* knockout (log_2_(Fold Change) at E6.5 = −0.22). Forkhead box protein A2 (*Foxa2*) encodes a protein belonging to a subfamily of the Forkhead box transcription factors and is an endoderm-specific gene [31]. There was no difference in expression levels of *Foxa2* and location of expression between wild-type and the *Sephs1^−/−^* embryo. We observed that only the rate of differentiation of the endoderm-originated organs, such as the digestive and respiratory systems, was retarded and these organs appeared to be at the E9.5 stage in the *Sephs1^−/−^* embryo. It appears that SEPHS1-deficiency does not affect the morphology of endodermal lineage tissues during development. Eomesodermin (*Eomes*), also referred to as T-box brain protein 2 (*Tbr2*), is a member of the T-box family of transcription factors initially expressed in the extraembryonic ectoderm and is known to play an important role in anterior visceral endoderm formation and epithelial-mesenchymal transition (EMT) [32,33]. In the wild-type embryo, *Eomes* was expressed throughout the extraembryonic ectoderm. However, in the *Sephs1^−/−^* embryo, the expression area was limited to the bottom of the extraembryonic ectoderm region, wherein the expression levels were slightly decreased (log_2_(Fold Change) at E6.5 = −0.15), suggesting that extraembryonic lineage tissues, such as the yolk sac and placenta, may differentiate abnormally after E6.5. In conclusion, expression levels and locations of the dermal layer marker genes provide evidence that knockout of *Sephs1* causes morphological changes as well as the retardation of development during dermal layer formation. 

### 2.3. Morphological Changes in the Embryonic Region by Sephs1 Knockout

**Figure 3:**
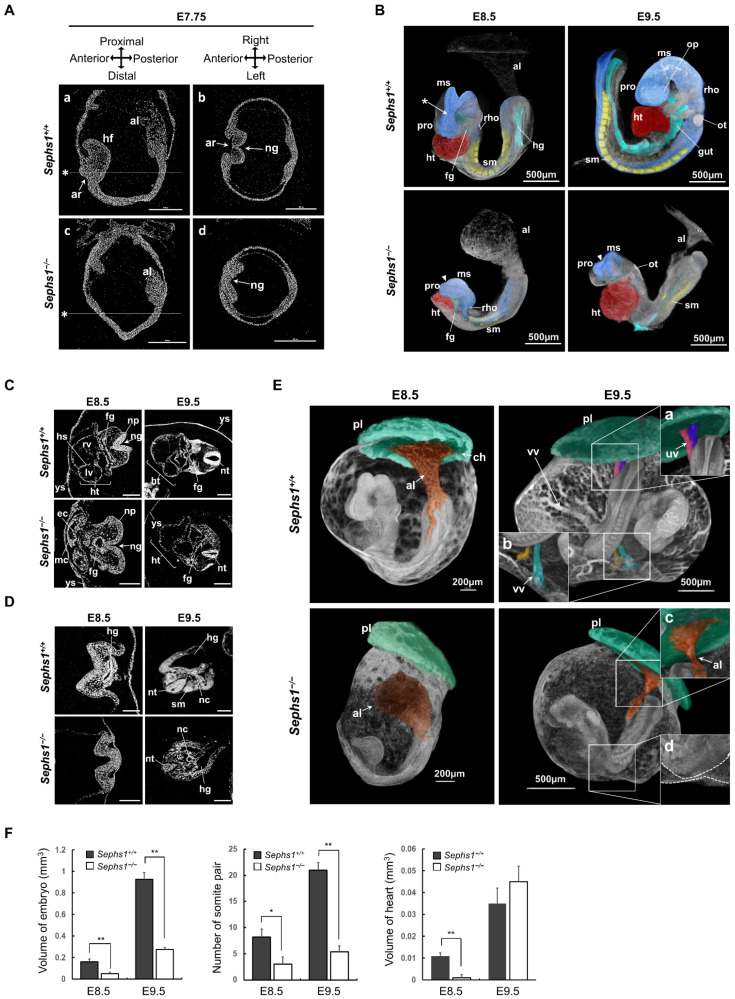
Embryonic defects in *Sephs1^−/−^* embryos. (**A**) Virtual view of embryos cut in sagittal (**a**,**c**) and transverse (**b**,**d**) directions at E7.75. Transverse views shown in b and d were generated at positions marked with an asterisk in a and c, respectively. Scale bars represent 200 µm. (**B**) 3-dimensional reconstruction of XRM data. Each organ was indicated by a different color using the Region of Interest tool in Dragonfly software (red, heart; yellow, somite; blue, neural plate; light blue, gut). (**C**,**D**) Transverse section of XRM data. The upper embryonic body (**C**) and the lower half of the embryonic body (**D**) were virtually sectioned. Scale bars represent 200 µm. (**E**) Comparison of detailed structure of allantois. The junction sites of the umbilical vein and vitelline vein are more magnified in the box. (**F**) Statistical analysis of 3-dimensional reconstruction. Volume was calculated by Dragonfly, ORS. * indicates *p* < 0.05. ** indicates *p* < 0.01. orange, allantois; green, chorion; pink and purple, umbilical vessel; blue and yellow, vitelline vessel. al, allantois; ar, archenteron; ch, chorion; ec, endocardium; fg, foregut; hg, hindgut; hs, heart sectum; ht, heart; mc, myocardium; lv, left ventricle; ms, mesencephalon; ng, neural groove; np, neural plate; nt, neural tube; ot, otic vesicle; pl, placenta; pro, prosencephalon; rho, rhombencephalon; rv, right ventricle; sm, somite; uv, umbilical vessel; vv, vitelline vessel; and ys, yolk sac.

We confirmed the developmental abnormalities predicted in Figure 2 by examining morphological changes in the *Sephs1^−/−^* embryos using X-ray microscopy (XRM) technology (Figure 3). The central nervous system of the *Sephs1^−/−^* embryo manifested retarded development and abnormal shape compared to those of the wild-type embryo. At E7.75, head fold (hf) was found in the wild-type embryo (panel (a) of Figure 3A) but not in the *Sephs1^−/−^* embryo (panel (c) in Figure 3A). At E8.5 and E9.5, although three primary brain vesicles (prosencephalon (pro) and mesencephalon (ms) and rhombencephalon (rho)) were formed, the size of brain tissue in the *Sephs1^−/−^* embryo was much smaller than that in wild-type, and the neural groove between prosencephalon (pro) and mesencephalon (ms) was not closed (arrowheads in Figure 3B). In the transverse section of the cervical region in E8.5, the closures of the neural grooves in wild-type embryos were progressing, whereas in the *Sephs1^−/−^* embryo, it was open to the outside (Figure 3C). The neural plate (blue) was closed around somites (yellow) in the wild-type embryo (Video S1), but remained open in the *Sephs1^−/−^* embryo (Video S2). In addition, the development of somites was delayed. Segmentation of somites was clearly observed in the wild-type embryo at E8.5 and E9.5, while somite segmentation did not occur in the *Sephs1^−/−^* embryo until E9.5 (see yellow-colored regions in Figure 3B). Segmented somites (yellow) were straight and parallel with the neural plate (blue), and clearly distinguished from the neural plate (blue) in wild type at E9.5 (Video S3). On the other hand, somites (yellow) in the *Sephs1^−/−^* embryo were severely twisted and had ambiguous boundaries (Video S4).

Heart development was also inhibited by SEPHS1-deficiency. As shown in Figure 3B, the differentiation of heart (ht) in the *Sephs1^−/−^* embryo at E8.5 proceeded slowly compared to wild-type. Heart structure of *Sephs1^−/−^* embryo remained in the cardiac crescent stage, while that in wild-type embryo developed into the heart tube stage showing ventricles and heart septum at E8.5 (Figure 3C). Notably, the size of the heart in both wild-type- and *Sephs1^−/−^* embryos was similar at E9.5, but the cell density was significantly reduced and the morphology of the heart was irregular in *Sephs1^−/−^* embryos (compare *Sephs1^+/+^* with *Sephs1^−/−^* of E9.5 in Figure 3C).

The archenteron (ar), which differentiates into foregut (fg) was not observed in the *Sepsh1^−/−^* embryo at E7.75 (Figure 3A), but observed at E8.5 and E9.5 (Figure 3C). Hindgut (hg) development seemed to proceed more slowly than foregut formation in the *Sephs1^−/−^* embryo. Hindgut formation was observed only at E9.5 in the *Sephs1^−/−^* embryo (Figure 3D). These data suggest that gut formation was not significantly affected by *Sephs1* knockout and this phenomenon is consistent with the result of FOXA2 expression patterns in Figure 2E. 

The process that connects allantois (al) with the chorion (ch) was completed in wild-type at E8.5, but al and ch were not connected in the *Sephs1^−/−^* embryo at E8.5 (Figure 3E). At E9.5, umbilical (uv) and vitelline vessels (vv, insets (a) and (b) in Figure 3E) were formed in wild-type, but not in the *Sephs1^−/−^* embryo (insets (c) and (d) in Figure 3E). The fact that the formation of vv and uv is inhibited by SEPHS1-deficiency suggests that SEPHS1 plays an essential role in the transport of substances between mother and embryo.

In addition, the total volume of the embryo was measured from the 3-dimensional structure obtained by reconstructing XRM images using a volume calculation software (Dragonfly). The volume ratio between the *Sephs1^−/−^* embryo and the wild-type decreased by approximately 3.2 times and 3.4 times at E8.5 and E9.5, respectively (Figure 3F). At E8.5, the number of somites (sm) in *Sephs1^−/−^* and wild-type embryos was 3 and 8.2, respectively. The gap in the somite number between *Sephs1^−/−^* embryo and wild-type embryos was increased at E9.5 (5.3 somites in the *Sephs1^−/−^* embryo and 21 somites in the wild-type embryo). The heart volume of the wild-type embryo at E8.5 was 10.1 times greater than that of the *Sephs1^−/−^* embryo. However, at E9.5, the heart volume in the *Sephs1^−/−^* embryo became abnormally enlarged (Figure 3C). Unlike the wild-type embryo, the *Sephs1^−/−^* embryo did not exhibit axial rotation at E9.5 and the embryonic axis was oriented in the same direction as at E8.5 (compare the E9.5 wild-type with the E9.5 *Sephs1^−/−^* embryos in Figure 3B). No further axial rotation was observed at E10.5 (data not shown) suggesting that axial rotation stopped at E8.5.

### 2.4. Pattern Analysis of DEGs Expressed in Extraembryonic Region

**Figure 4:**
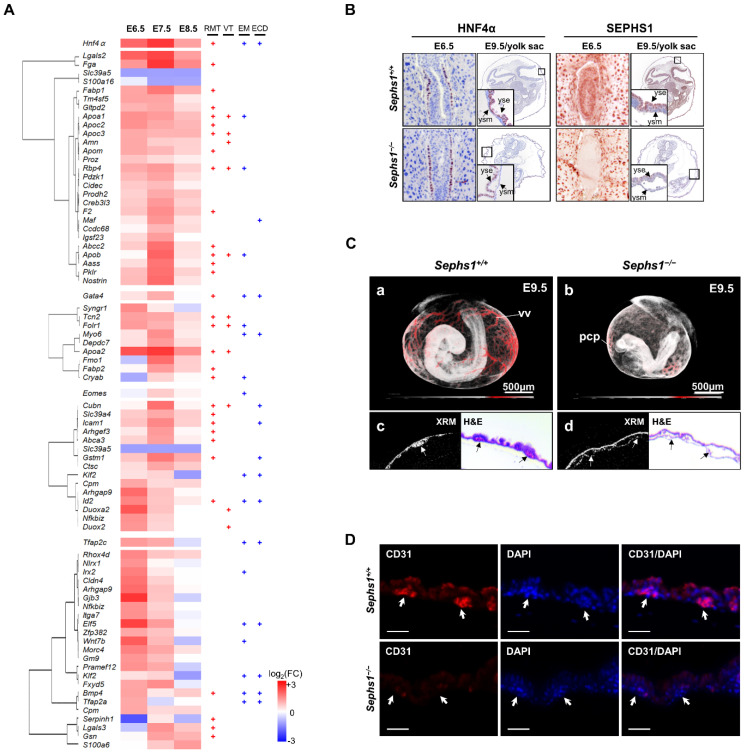
Expression patterns of transcription factors and its target DEGs in extraembryonic region and extraembryonic defects in *Sephs1^−/−^* embryo. (**A**) Expression pattern of *Hnf4α*, *Gata4*, *Eomes*, *Tfap2c*, and their target genes. Target genes obtained from the Chip-Atlas were filtered by expression specificity to the extraembryonic region. Among these genes, only DEGs were selected and subjected to hierarchical clustering. Target gene sets were subjected to GO analysis. On the right of the heat map, the GO term(s) assigned by each gene was (were) marked with ‘+’. RMT, Retinoid metabolism and transport; VIT, Vitamin transport; EM, Embryo morphogenesis; ECD, Epithelial cell differentiation. (**B**) Expression patterns of HNF4α and SEPHS1. Immunohistochemistry was performed against HNF4α and SEPHS1 as described in Materials and Methods. Small box was enlarged to inbox showing the yolk sac layer. (**C**) 3D structure of E9.5 embryos reconstructed from XRM images as described in Materials and Methods (**a**,**b**). The virtual section (XRM) and H&E staining image of paraffin section at the yolk sac (**c**,**d**). Arrow indicates hemato-endothelial progenitors. Scale bars represent 500 µm. (**D**) CD31 expression on yolk sac at E9.5. The images were acquired with a confocal microscope. Scale bars represent 50 µm. pcp, primary capillary plexus; vv, vitelline vessel; yse, and yolk sac ensoderm; ysm, yolk sac mesoderm.

Since biological processes (for example, ‘placenta development’ in Figure 2D) occurring in the extraembryonic region were also predicted, we hypothesized that development of the extraembryonic region also would proceed abnormally by SEPHS1 deficiency. To test if there is any abnormality of development in the extraembryonic region in the *Sephs1^−/−^* embryo, pathways involved in the development of extraembryonic region were identified and the pathway-related morphological changes were examined. We first selected DEGs among targets of extraembryonic region-specific DTFGs listed in Figure 2C. Four DTFGs expressed in the extraembryonic region and 70 of their target DEGs were identified. GO analysis was performed for the identified genes (Figure 4A). As a result, four pathways (Retinoid metabolism and transport (RMT), Vitamin transport (VT), Embryonic morphogenesis (EM), and Epithelial cell differentiation (ECD)) were predicted with high probability (log_10_(p) < −4.9).

*Hnf4α* and target genes are predicted to participate mainly in ‘Retinoid metabolism and transport’ and ‘Vitamin transport’ (RMT and VT in Figure 4A). Most of the genes in ‘Vitamin transport’ are included in ‘Retinoid metabolism and transport’ except *Duox2*, *Duoxa2*, *Cubn*, and *Amn*. *Duox2* is target of retinoic acid signaling and *Duoxa2* is a maturation factor of *Duox2* [34]. *Cubn*, which is the cobalamin (vitamin B12) receptor gene, has an inhibitory function against retinoic acid signaling and *Amn* encodes the protein that facilitates uptake of vitamin B12. Therefore, genes in ‘Vitamin transport’ are related with retinoic acid signaling and can be categorized into the same group with ‘Retinoid metabolism and transport’. It should be noted that GO analysis using total DEGs by IPA also predicted the retinoic acid signaling with the highest probability (Table S2). Retinoic acid is a morphogen participating in axis formation, embryo growth, and cell fate determination during early embryogenesis [35]. In addition, *Hnf4α* and its target genes are expressed in extraembryonic endoderm which develops into the yolk sac at a later stage suggesting that HNF4α plays an essential role in yolk sac development and its function via retinoic acid signaling.

*Gata4* and *Eomes*, and their target genes did not show enrichment in any specific pathway. Instead, they commonly participate in the pathways regulated by both *Hnf4α* and *Tfap2c*. It should be noted that *Gata4* is an activator of *Hnf4α* and is expressed more widely than *Hnf4α* [36]. 

*Tfap2c* and target genes are predicted to participate mainly in embryo morphogenesis and epithelial cell differentiation (EM and ECD in Figure 4A). During organogenesis, embryonic morphogenesis and epithelial cell differentiation should occur together. Therefore, embryo morphogenesis and epithelial cell differentiation can be categorized into organogenesis. In addition, both embryo morphogenesis and epithelial cell differentiation share BMP4 as a key protein suggesting BMP4 is used as a common morphogen for embryo morphogenesis and epithelial cell differentiation. Recently, it was reported that *Tfap2c*, which is expressed in extraembryonic ectoderm, plays an essential role in the development of trophoblast and placenta [37]. Chorioallantoic fusion is a process in trophoblast differentiation. As described in the previous section, we found chorioallantoic fusion did not occur in the *Sephs1^−/−^* embryo (Figure 3E) suggesting that *Tfap2c* and its target genes inhibit chorioallantoic fusion in the SEPHS1-deficient embryo.

Since *Hnf4α* is known to be expressed in extraembryonic endoderm, we examined the region where this gene was expressed during gastrulation. At E6.5, HNF4α was distributed more widely in the extraembryonic region of the *Sephs1^−/−^* embryo than that of wild-type, and at E9.5, the cell density of HNF4*α*-expressing yolk sac endoderm was lower in the *Sephs1^−/−^* embryo than that of the wild-type (Figure 4B). The yolk sac endoderm morphology was concavo-convex in the wild-type but became flat in the *Sephs1^−/−^* embryo. Another significant difference in the structure of the yolk sac between wild-type and *Sephs1^−/−^* embryo was that the yolk sac endoderm and mesoderm were separated in the *Sephs1^−/−^* embryo at E9.5. XRM image showed that vitelline vessels (vv), which are normally generated in the yolk sac at E9.5 stage, were not found in the *Sephs1^−/−^* embryo (compare panels (a) and (b) of Figure 4C). Separation of the yolk sac endoderm and mesoderm was also observed in the *Sephs1^−/−^* embryo (panel (d) of Figure 4C). At the same time point, expression of CD31, which is known as a hemato-endothelial progenitor marker [38], was not detected in the *Sephs1^−/−^* embryo, while it was detected in the wild-type embryo suggesting that both vitelline vessel and blood progenitors were not formed due to SEPHS1 deficiency (Figure 4D).

It should be noted that SEPHS1, which is known to be expressed in all tissues, was expressed only in yolk sac endoderm, not yolk sac mesoderm in the wild-type embryo (Figure 4B) suggesting that SEPHS1 is expressed cell-type specifically in the same tissue and its deficiency affects mainly the function of yolk sac endoderm at late gastrulation.

### 2.5. Pathway Prediction through Protein–Protein Interaction and Gene Set Enrichment Analysis

**Figure 5:**
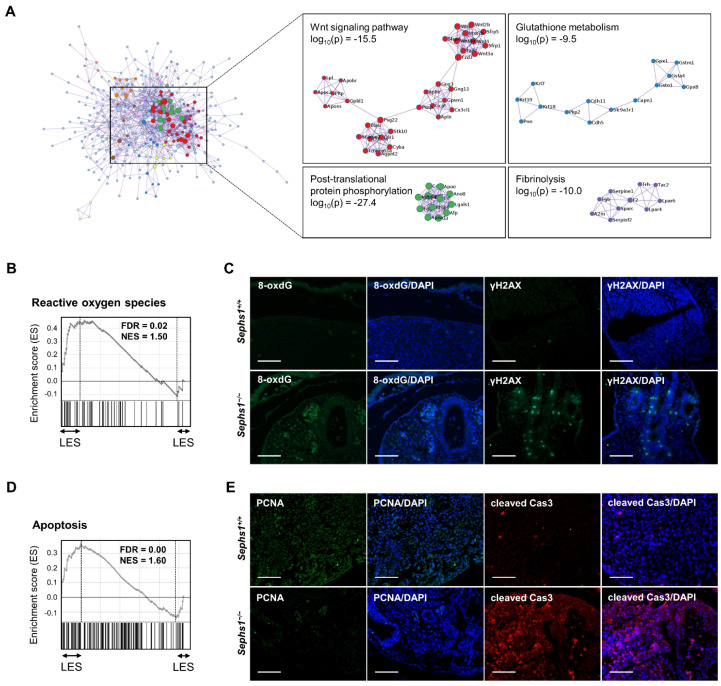
*Sephs1^−/−^* embryo shows growth retardation, apoptosis and ROS stress. (**A**) Protein–protein interaction map at E6.5 using PPI analysis tool plugged-in Metascape. (**B**) Enrichment score plot of Reactive oxygen species obtained by gene set enrichment analysis (GSEA). The ranked list was created by sorting the gene list according to fold-change. LES, Leading edge subset. (**C**) Immunohistochemistry of 8-oxo-guanine and γH2AX. (**D**) Enrichment score plot of Apoptosis obtained by GSEA. The ranked list was created as described above. (**E**) Protein level of PCNA and cleaved caspase 3. Immunohistochemistry was performed as described in Materials and Methods. The images were acquired with an Axiovert 200M inverted microscope. Scale bars represent 100 µm.

In order to identify the signaling pathway and molecular mechanism that participate in gastrulation, additional analyses were performed. By analyzing protein–protein interaction (PPI) of DEGs at E6.5, we could obtain three modules with high probability (log_10_(p) < −9.5); Wnt signaling pathway, Glutathione metabolism, and the post-translational protein phosphorylation pathway (Figure 5A).

Wnt signaling regulates mainly morphogenesis, such as growth in the early embryo, axis formation, and pattern formation [39]. Wnt signaling was inhibited in *Sephs1*^−/−^ embryos at E6.5, suggesting that pathways determining embryonic morphology were affected by *Sephs1* knockout before the organogenesis stage. Glutathione is one of the molecules that plays an essential role in maintaining redox homeostasis. Imbalance in glutathione metabolism by *Sephs1* knockout leads to the accumulation of ROS that causes oxidative stress in the cell [19]. Prediction of post-translational protein phosphorylation with high probability suggests that signaling pathways are actively regulated, since almost all signaling pathways are regulated through phosphorylation of proteins participating in each cognate pathway. These results of PPI analysis suggest that SEPHS1 deficiency causes oxidative stress by disrupting redox homeostasis, and that the oxidative stress will primarily affect Wnt signaling.

Besides GO analysis, another useful method for pathway prediction is Gene Set Enrichment Analysis (GSEA). Because GO analysis uses only a gene list to predict pathways or processes without giving weight according to fold change value of each gene, we cannot determine to what extent a specific gene contributes to those pathways or processes. The advantage of GO analysis is that one can identify all possible pathways or processes enriched by DEGs. On the other hand, with GSEA methods, we can determine how a specific gene contributes to a specific pathway or process, since DEGs are ranked according to their fold change value and the ranked DEGs are used to calculate enrichment score for a pathway or process of interest [40].

GSEA with total genes using the Hallmark Gene Sets from the Molecular Signatures Database revealed that ‘Reactive Oxygen Species’ was predicted with high significance where normalized enrichment score (NES) and false discovery rate (FDR) were 1.50 and 0.02, respectively (Figure 5B). There are 28 genes within the leading-edge subset (LES) of ‘Reactive Oxygen Species’ (Table S3). The genes include *Glrx1*, *Prdx2*, *Prdx6*, *Txnrd1*, *Txnrd2*, and *Nqo1*, and these genes participate in redox homeostasis or ROS generation. We then examined the gene number within LES and NES of ‘Reactive Oxygen Species’ in more detail at each embryonic day. The gene numbers within LES were 25, 27, and 31 at E6.5, E7.5, and E8.5, respectively (Table S3). In addition, the NES of ‘Reactive Oxygen Species’ at E6.5, E7.5, and E8.5 were 0.56, 1.0, and 1.42, respectively (Figure S2B). These results suggest that ROS levels in the embryo are gradually increased. DNA damage, such as formation of γH2AX and 8-oxoguanine, is commonly used as an oxidative stress marker. 

In order to determine whether DNA damage due to oxidative stress occurred in the *Sephs1* knockout, immunohistochemistry (IHC) using an antibody against 8-oxoguanine and γH2AX (phosphorylated H2AX) was performed. Neither formation of γH2AX nor of 8-oxoguanine were detected from E6.5 to E8.5 (Figure S2C) in *Sephs1^+/−^* or *Sephs1^−/−^* embryos. However, both 8-oxoguanine and γH2AX were generated only in the *Sephs1^−/−^* embryo at E9.5 (Figure 5C). These results indicate that oxidative stress was too mild to cause DNA damage in *Sephs1^−/−^* embryos by E8.5, but was strong enough to cause DNA damage at E9.5.

In addition to ‘Reactive Oxygen Species’, ‘Apoptosis’ was also predicted with high significance where NES and FDR were 1.6 and 0.00, respectively (Figure 5D). The leading-edge subset of ‘Apoptosis’ consists of 63 genes (Table S4) including *Sqstm1*, *Pdcd4*, *Smad7*, *Hspb1*, *Faslg*, *Bax*, *Madd*, *Bmf*, and *Hgf*, which are known to participate in apoptotic signaling. The gene numbers within LES and the NESs were gradually increased as embryogenesis proceeded (Figure S2B and Table S4). Unexpectedly, we could not detect the activated form of caspase-3 in SEPHS1-deficient embryos until E8 (Figure S2D). However, the activated form of caspase-3 was detected in the *Sephs1^−/−^* embryo at E9.5, suggesting that SEPHS1 deficiency induced cell death through apoptosis in the embryo (Figure 5D). Notably, genes such as *Gadd45b*, *cdkn1a*, and *Btg2*, which are related to cell proliferation, were also included in the apoptosis pathway. Therefore, we assumed that cell proliferation was also inhibited by *Sephs1* knockout. PCNA is used as the most reliable marker for evaluating cell proliferation. As shown in Figure 5E, the level of PCNA was significantly decreased in the *Sephs1^−/−^* embryo. These results strongly suggest that SEPHS1-deficiency causes inhibition of cell proliferation followed by apoptosis.

## 3. Discussion

SEPHS1 regulates various cellular functions such as redox homeostasis, and thus is known to be essential for embryo survival and growth. However, the detailed mechanisms of which genes or pathways are controlled by *Sephs1* during development and how they result in embryonic lethality have not been fully elucidated. Through transcriptome analysis, the pathways that are responsible for morphological defects in *Sephs1^−/−^* embryo were predicted. To predict pathways affected by *Sephs1* knockout, a combination of various bioinformatic tools (AmiGO and Metascape analysis, IPA analysis and GSEA) with various databases for protein–protein interaction (String and BioGrid), transcription factor (TRRUST and ChIP-Atlas) and cell type (Mouse Gastrulation Atlas and MGI) were applied.

Bioinformatic analyses suggested several interesting features of the effect of SEPHS1-deficiency on the development at early embryonal stages. Throughout the gastrulation stage, coagulation was predicted as the most highly affected pathway (log_10_(p) = −11.9). Genes included in this GO term are implicated in cell migration and adhesion which are the most prominent features of cells involved in the dermal layer and in axis formation [41]. Among signaling pathways participating in gastrulation, retinoic acid signaling was predicted to be activated throughout gastrulation with high significance (log_10_(p) = −8.9). Retinoic acid is a morphogen, and its signaling pathway is known to regulate axis formation and cell fate determination [35]. The upregulation of *Rbp4*, which mediates the transport of retinoic acid into the cell suggests that the levels of intracellular retinoic acids were increased by *Sephs1* knockout. In addition, we found that the targets of retinoic acid pathway were also increased in their expression levels, suggesting that retinoic acid signaling was hyperactivated. Hyperactivation of retinoic acid signaling, due to imbalance of retinoic acid concentration in the cells, may adversely affect embryonal axis formation and cell fate determination. We observed various abnormalities during gastrulation and early organogenesis of SEPHS1-deficient embryo, including the absence of axial rotation.

PPI analysis produced other interesting findings. Wnt, prolactin and insulin-like growth factor (IGF) signaling pathways were predicted to be affected by *Sephs1* knockout at E6.5, E7.5, and E8.5, respectively (Figure 5A and Figure S2A). Wnt signaling is known to be involved in regulation of growth in early embryo development, axis formation, and pattern formation [39,42]. Since the expression of *Wnt3a*, *Wnt2b*, *Wnt8a*, and *Fzd3* were significantly decreased in *Sephs1^−/−^* embryo at E6.5, Wnt signaling seemed to be inhibited. Among the direct targets of Wnt signaling listed in the database ‘the Wnt’ [43], the expression of 85% of the targets was down-regulated in the *Sephs1^−/−^* embryos. At E6.5, *Sephs1^−/−^* embryos showed changes in the expression level and location of dermal layer markers, such as *Sox*, *Otx2*, and *Eomes*, which may lead to the retardation of embryonic growth and axis formation at E7.5 (see Figure 1A and Figure 2E). Prolactin is known to play an important role in the trophoblast growth and the development and differentiation of neural crest cells in neurulation stage, but its role in the gastrulation stage has not been clearly identified [44]. Since the expression of *prl2c2*, *prl2c3*, *prl4a1*, and *prl5a1* was increased in the *Sephs1^−/−^* embryos, we hypothesized that prolactin signaling was hyperactivated. Among the target genes of the prolactin pathway in KEGG DB (map04917; Prolactin signaling pathway), the expression of *Socs3*, *Elf5*, *Prlr*, and *Slc2a2* were increased by more than 1.5-fold in the *Sephs1^−/−^* embryos at E7.5. Interestingly, although not at E7.5, abnormal development of the central nervous system was observed at E8.5 (Figure 3B,C). One of the important roles of prolactin is to control the development of the nervous system [44]. IGF signaling is known to be involved in promoting growth of embryos and organ development [45]. The expression of IGFBP3 and other proteins including proteases that binds to IGFBP3 were up-regulated in the *Sephs1^−/−^* embryos (Figure S2A). Binding of IGF to IGFBP3 and binding of IGFBP3 to proteases inhibit IGF signaling [46]. Therefore, SEPHS1-deficiency seems to inhibit IGF signaling during late gastrulation and early neurulation. The *Sephs1^−/−^* embryos showed growth retardation, structural brain abnormalities and disrupted cardiac development at E8.5 and these phenotypes are consistent with those described in another study [45]. These results suggest that SEPHS1-deficiency affects embryo morphogenesis by regulating Wnt and retinoic acid signaling and then organogenesis by regulating retinoic acid, prolactin, and IGF signal pathways in combination.

It should be noted that most of the genes responsible for reception of retinoic acid signaling (apolipoprotein and retinoic acid binding protein genes) are expressed in the extraembryonic region specifically, but its target genes are expressed both in the embryonic and extraembryonic regions. This finding is consistent with the experimental results. For example, both vitelline vessel formation and chorioallantoic fusion did not occur in the *Sephs1^−^^/^^−^* embryo (Figure 3 and Figure 4). Abnormalities in the yolk sac and placental development will inhibit nutrient uptake and waste disposal from the embryo.

**Figure 6:**
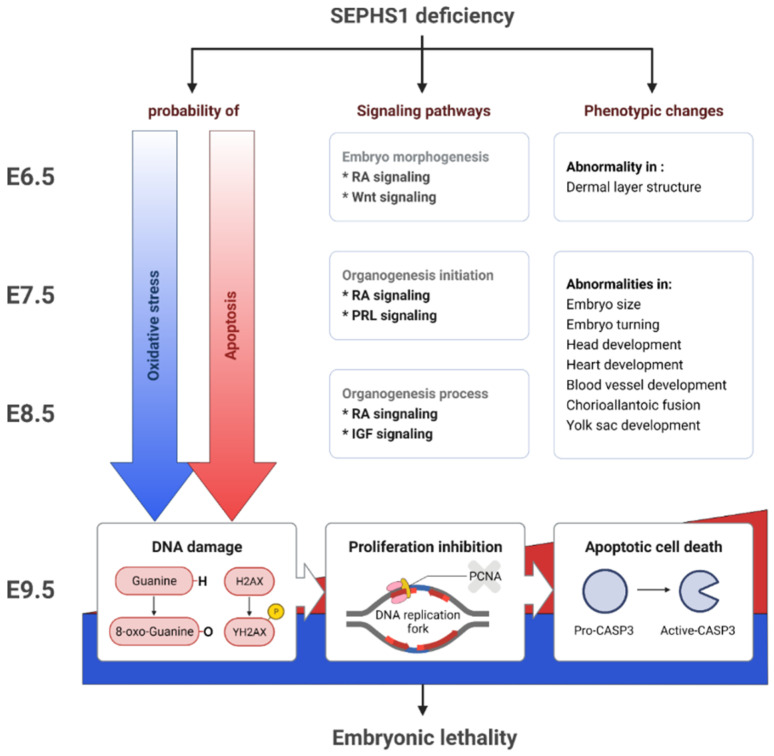
Schematic diagram of effect of SEPHS1 deficiency on early embryogenesis.

Taking the results from both the bioinformatic analyses and experimental evidence into consideration, we propose the role of SEPHS1 during early mouse embryogenesis as follows (Figure 6). SEPHS1 regulates intracellular ROS levels through regulating the redox homeostasis system. The deficiency of SEPHS1 causes disruption of the redox homeostasis system and leads to a gradual increase in ROS levels. Low levels of ROS will cause mild oxidative stress, leading to abnormalities in signaling pathways. During early gastrulation, abnormalities in embryonic morphogenesis, such as dermal layer and axis formation, occur presumably through Wnt and retinoic acid signaling. Abnormalities in organogenesis occur presumably through prolactin and retinoic acid signaling, followed by insulin-like growth hormone and retinoic acid signaling during mid- and late-gastrulation, respectively. ROS accumulate sufficiently to cause cell death through DNA damage at E9.5, followed by embryonic death. Dead embryos then undergo resorption by E14.5 [19]. Since the signaling pathways were predicted using bioinformatic tools, future molecular studies will be needed to further validate our results.

In this study, we elucidated how SEPHS1-deficiency induces developmental abnormality and embryonic lethality. Our results provide evidence that SEPHS1-deficiency is one of the contributors of natural miscarriages, and that the levels of SEPHS1 can be used as a marker for diagnosis or prognosis of natural miscarriages.

## 4. Materials and Methods

### 4.1. Materials

Antibodies against SEPHS1, γH2AX, 8-oxo-guanine, POU5F1, CFL488-conjugated mouse IgG, and CFL488-conjugated rabbit IgG were purchased from Santa Cruz Biotech (Dallas, TX, USA). Antibodies against CD31, EOMES, FOXA2, HNF4α, cleaved caspase3, PCNA and cy3 conjugated rabbit IgG, AEC substrate, and Mayer’s modified hematoxylin were purchased from Abcam (Cambridge, UK). Antibody against SOX2 and OTX2 were purchased from R&D Systems (Minneapolis, MN, USA). Biotin-conjugated antibody against mouse IgG and rabbit IgG were purchased from Jackson Immunoresearch (West Grove, PA, USA). MG^TM^ Tissue SV kit was purchased from MG Med (Seoul, Korea). Trizol^TM^ reagent, DNase I, PowerUp^TM^ SYBR^TM^ Green Master Mix were purchased from Thermo Fisher (Waltham, MA, USA). MMLV-RT was purchased from Enzynomics (Daejeon, Korea). TruSeq RNA Sample Prep Kit v2 was purchased from Illumina (San Diego, CA, USA). Lugol’s solution, streptavidin-HRP, DAPI and paraformaldehyde were purchased from Sigma-Aldrich (Burlington, MA, USA). Slide glasses coated with 3-aminopropyl triethoxysilane was purchased from Matsunami Glass (Osaka, Japan).

Programs and their websites are as follows:

AmiGO [47]: http://amigo.geneontology.org/amigo

Ballgown [48], DESeq2 [49] and edgeR [50]: plugged in R: https://www.r-project.org/

BioRender (Toronto, Ontario, Canada): Figure 6 was created with https://biorender.com/

ChIP-Atlas [51]: https://chip-atlas.org/

Dragonfly (ORS, Montreal, Quebec, Canada): https://www.theobjects.com/index.html

FastQC (Babraham Bioinformatics, Babraham Institute, Cambridge, UK): https://www.bioinformatics.babraham.ac.uk/projects/fastqc

GenePattern 2.0. [52]: https://www.genepattern.org/

Gene Set Enrichment Analysis [40]: https://www.gsea-msigdb.org/gsea/index.jsp

HISAT2 [53]: http://daehwankimlab.github.io/hisat2/

Ingenuity Pathway Analysis (QIAGEN, Germantown, MD, USA): http://www.ingenuity.com

Metascape [54]: https://metascape.org/gp/index.html

Mouse Gastrulation Atlas [55]: https://marionilab.cruk.cam.ac.uk/MouseGastrulation2018/

Mouse Genome Informatics (MGI) [56]: http://www.informatics.jax.org/index.shtml

MsigDB [57]: https://www.gsea-msigdb.org/gsea/msigdb/

PANTHER [58]: http://www.pantherdb.org/

PRISM (GraphPad Software, San Diego, CA, USA): https://www.graphpad.com/scientific-software/prism/

StringTie [59]: https://ccb.jhu.edu/software/stringtie/

TreeView [60]: http://jtreeview.sourceforge.net/

Trim Galore (Babraham Bioinformatics, Babraham Institute, Cambridge, UK): https://www.bioinformatics.babraham.ac.uk/projects/trim_galore/

TRRUST v2 [61]: https://www.grnpedia.org/trrust/

### 4.2. Generation of SEPHS1 Total Knockout (Sephs1^−/−^) Mice

All mice used in this study were on a C57BL/6J background. The generation of SEPHS1 total knockout (*Sephs1^−/−^*) mice have been described previously [19]. Female *Sephs1*^+/−^ mice were crossed with male *Sephs1^+/−^* mice, and then dissected to obtain *Sephs1^−/−^* embryos. Embryonic day (days post-coitus) E0.5 was defined as noon on the day that a mating plug was detected. We used 32 embryos (25 *Sephs1^+/^^−^* and 7 *Sephs1^−/−^*), 24 embryos (17 *Sephs1^+/−^* and 7 *Sephs1^−/−^*), 34 embryos (26 *Sephs1^+/−^* and 8 *Sephs1^−/−^*), 20 embryos (16 *Sephs1^+/−^* and 4 *Sephs1^−/−^*), 17 embryos (14 *Sephs1^+/−^* and 3 *Sephs1^−/−^*), and 10 embryos (8 *Sephs1^+/−^* and 2 *Sephs1^−/−^*) for morphological observation and histological analysis at E6.5, E7.5, E8.5, E9.5, E10.5 and E11.5, respectively. All procedures performed involving the mice were conducted in accordance with the Institutional Guidelines of the Institute of Laboratory Animal Resources (Seoul National University, Seoul, Korea). All mouse experiments were approved by the Institutional Animal Care and Use Committee at Seoul National University. All mice used in this study were bred and cared for under sterile conditions with constant temperature and humidity in the specific pathogen free animal facility at Seoul National University.

### 4.3. Genotyping

For genotyping using DNA, genomic DNA was extracted using the MG Genomic DNA Purification kit following the manufacturer’s instruction and then subjected to PCR. The wild-type (WT) allele was amplified by gWT genotyping primer set and the knockout (KO) allele was amplified by gKO genotyping primer set. For genotyping using RNA, total RNA was extracted with Trizol^TM^ reagent following the manufacturer’s instruction. Total RNA was reverse-transcribed by MMLV-RT and then cDNA was subjected to PCR. The wild-type (WT) allele was amplified by cWT genotyping primer set and the KO allele was amplified by cKO genotyping primer set. Primer sequences are as follows:

gWT_genotype, forward: 5′-GAGATGCGTTTGTGTCCTCC-3′

gWT_genotype, reverse: 5′-AGTGAGTGCCCGCCTTTA-3′

gKO_genotyping, forward: 5′-GTGTCCTCCATAACTTCGTATAGC-3′

gKO_genotyping, reverse: 5′-GAGAGCAGCAGTGTAGAGGTC-3′

cWT_genotype, forward: 5′-GAGAGTCCTTTAACCCGGAG-3′

cWT_genotype, reverse: 5′-AGGAAAGACCACCATGCCTC-3′

cKO_genotyping, forward: 5′-ATTCAGGAGACGCTTAAGG-3′

cKO_genotyping, reverse: 5′-AGGAAAGACCACCATGCCTC-3′

For genotyping using paraffin-embedded sections, immunohistochemistry against anti-SEPHS1 antibody was performed.

### 4.4. Embryo Preparation and RNA Extraction for RNA-seq

Embryos were dissected from the uterus at E6.5, E7.5 and E8.5 with the aid of super-fine forceps and a 29-gauge needle. RNA was extracted from whole embryos using Trizol^TM^ reagent with glycogen carrier. After genotyping, *Sephs1^+/+^*, *Sephs1^+/−^*, or *Sephs1^−/−^* embryos were pooled separately according to their genotype. To remove genomic DNA (gDNA), RNA samples were treated with DNase I following the manufacturer’s instruction. mRNA quality was assessed using the RNA 6000 Nano-Assay on a BioAnalyser 2100 (Agilent Technologies, Waltham, MA, USA). A total of 118 embryos at E6.5 (30 *Sephs1^+/+^*, 63 *Sephs1^+/^^−^*, and 25 *Sephs1^−/−^* embryos), 86 embryos at E7.5 (21 *Sephs1^+/+^*, 39 *Sephs1^+/−^*, and 26 *Sephs1^−/−^* embryos), and 25 embryos at E8.5 (6 *Sephs1^+/+^*, 15 *Sephs1^+/−^*, and 4 *Sephs1^−/−^* embryos) were used.

### 4.5. Realtime PCR

Realtime PCR was performed as described previously [19] with minor modifications. Total RNA isolated using Trizol^TM^ reagent was reverse-transcribed by M-MLV reverse transcriptase, and then subjected to real-time PCR using PowerUp^TM^ SYBRTM Green Master with the StepOne^TM^ system (Applied Biosystems, Waltham, MA, USA) according to the manufacturer’s instructions. Primer sequences are listed below:

Pou5f1 (Oct4), forward: 5′-AAGTTGGCGTGGAGACTTTG-3′

Pou5f1 (Oct4), reverse: 5′-CCGCAGCTTACACATGTTCT-3′

Nanog, forward: 5′-CGGCTCACTTCCTTCTGACT-3′

Nanog, reverse: 5′-GCGTTCCCAGAATTCGATGC-3′

Brachyury (T), forward: 5′-CTGTGAGTCATAACGCCAGC-3′

Brachyury (T), reverse: 5′-AGATCCAGTTGACACCGGTT-3′

Six3, forward: 5′-TTGCTCTCTCTAACTCGCTGG-3′

Six3, reverse: 5′-CCCGACCCTTGTTCATCTGG-3′

### 4.6. Transcriptome Analysis

Transcriptome analyses were performed using the TruSeq RNA Sample Prep Kit v2 and paired-end sequencing (Illumina HiSeq 4000). We acquired 101-bp reads in 6.3–7.4 Gb per sample. Data yield was approximately 7.0 × 10^7^ raw reads, and approximately 98.5% reads were mapped. Sequencing quality was assessed by FastQC and low-quality bases were trimmed using Trim Galore. Alignment of reads to the mouse reference genome (mm10) was carried out by HISAT2 and STAR aligner. Gene quantification was performed by StringTie, gene counts by HTseq-count and differential expression analysis by Ballgown and DESeq2 packages in R. Differential gene expression was evaluated using max (FPKM) > 1, *p* < 0.01 and |log_2_(Fold Change)| > 1. To reduce the sample-to-sample systematic bias that may affect the interpretation, the data were calibrated by Trimmed Mean of M-values (TMM) normalization and estimating the size factor using count data ‘edgeR’ in R.

### 4.7. Pathway Analyses and Transcription Factor Prediction

Ingenuity Pathway Analysis (IPA) was carried out using the canonical pathway module in IPA and Gene Set Enrichment Analysis (GSEA) using pre-ranked modules. Functional enrichment analysis was carried out using the specified gene lists by PANTHER or Metascape. Transcription factor analysis was performed using TRRUST and PaGenBase modules in Metascape. Protein–protein interaction analysis was carried out using BioGrid and String modules in Metascape.

Manual transcription factor analysis was performed using TRRUST database. All transcription factors and their target lists were recruited from the TRRUST database, and then filtered into the ‘gastrulation’ GO category. The ratio of DEGs among these target genes was calculated. Transcription factors were filtered with max (FPKM) > 10, the number of target gene (s) in ‘gastrulation’ GO category > 1 and DEGs in target gene (%) > 50%.

Hierarchical clustering was performed using GenePattern. Distance was calculated by Euclidean distance or Pearson correlation, and clustered by pairwise complete-linkage method. Heatmap was illustrated by Treeview.

### 4.8. X-ray Microscopy Imaging

#### 4.8.1. Sample Preparation

Embryos were prepared, stained, and imaged as described previously with slight modification [62]. Embryos were dissected within decidua to fully preserve embryo to extra-embryonic association without disturbing the orientation or morphology. Embryos were fixed in 4% paraformaldehyde overnight at 4 °C and were washed with 1X PBS three times for 1 h each. Fixed samples were immersed in 0.1 N (*v*/*v*) Lugol’s solution at room temperature for varying time according to the size of the embryo, 3–5 days. After staining, samples were mounted individually within a microcentrifuge tube filled with 0.5% *w*/*v* agarose and imaged immediately.

#### 4.8.2. Image Acquisition

The raw data for 3D imaging of the samples were acquired via Xradia 620 versa (Carl Zeiss, Oberkochen, Baden-Württemberg, Germany). Each data set was acquired with the X-ray source at 60 kV and 142 µA with a 0.5 mm aluminum attenuation filter. The acquisition time/isotropic voxel is 2 h/1.3 voxel to 4 h/0.8 voxel. Each sample was rotated 360° along the anterior–posterior axis, and a projection image at 2016 × 1344 pixels was generated every 0.3° at an average of 4 images. Acquired projection images were reconstructed and analyzed via Dragonfly (Version: 2021.1; ORS, Montreal, Quebec, Canada) software.

### 4.9. Histological Analysis

Embryos were dissected and fixed with 4% paraformaldehyde overnight at 4 °C. Fixed embryos were washed in running tap water for at least 6 hr and then paraffin embedded. The paraffin embedding process was performed automatically by Tissue processor (Leica, Wetzlar, Germany) following the routine overnight protocol. Paraffin blocks were sliced to 5 μm thickness, and then the slices attached to slide glasses coated with 3-aminopropyl triethoxysilane. After overnight drying at 37 °C on a slide warmer, the sample slides were used for further analysis. Hematoxylin and eosin staining was processed automatically by Autostainer (Leica).

### 4.10. Immunohistochemistry

Immunohistochemistry was performed following standard protocols using primary antibodies: anti-SEPHS1 (1:100), anti-EOMES (1:200), anti-HNF4α (1:100), anti-FOXA2 (1:200), anti-SOX2 (1:200), anti-OTX2 (1:100), anti-8-oxo-guanine (1:100), anti-γH2AX (1:100), anti-cleaved caspase 3 (1:100), anti-PCNA (1:200), and anti-CD31 (1:100). Briefly, sample slides were incubated with xylene for paraffin removal and ethanol for rehydration. Antigen retrieval was performed with sodium citrate (pH 5.0) or Tris-EDTA (pH 9.0) buffer, according to the manufacturer’s instruction for the primary antibody. Slides were blocked with 5% normal goat serum in Tris-buffered saline/Tween for 1 h at room temperature. After the incubation with primary antibody overnight at 4 °C, CFL488-conjugated mouse IgG, Cy3-conjugated rabbit IgG, biotin-conjugated mouse IgG or biotin-conjugated rabbit IgG was applied for 1 h at room temperature, and biotin-conjugated IgGs were incubated with streptavidin-HRP for 1 h at room temperature. Slides were counter-stained with DAPI and observed using a fluorescence microscope (Axiovert 200M, Carl Zeiss) or confocal microscope (LSM710, Carl Zeiss). In the case of chromogen dye, color development was performed using an AEC substrate and samples were counter-stained with Mayer’s modified hematoxylin. Slides were observed using a stereo microscope (SMZ18, Nikon, Tokyo, Japan).

### 4.11. Statistical Analysis

Statistical analyses were performed using an unpaired Student’s *t*-test or one-way ANOVA with GraphPad PRISM 7.0. Statistical analysis for DEGs were performed by exact test using edgeR. A value of *p* < 0.01 was considered significant.

## Data Availability

All of the raw datasets can be found in the Short Read Archive (SRA) database of the National Center for Biotechnology Information (NCBI) under accession number PRJNA764535.

## Supplementary Materials

The following are available online at https://www.mdpi.com/article/10.3390/ijms222111647/s1.

## References

[1] Brigelius-Flohé R., Sies H. (2016). Diversity of Selenium Functions in Health and Disease.

[2] Gladyshev V.N., Hatfield D.L., Schweizer U., Tsuji P.A., Hatfield D.L. (2016). Selenium: Its Molecular Biology and Role in Human Health.

[3] Na J., Jung J., Bang J., Lu Q., Carlson B.A., Guo X., Gladyshev V.N., Kim J.-H., Hatfield D.L., Lee B.J. (2018). Selenophosphate synthetase 1 and its role in redox homeostasis, defense and proliferation. Free. Radic. Biol. Med..

[4] Ghaemi S.Z., Forouhari S., Dabbaghmanesh M.H., Sayadi M., Bakhshayeshkaram M., Vaziri F., Tavana Z. (2013). A Prospective Study of Selenium Concentration and Risk of Preeclampsia in Pregnant Iranian Women: A Nested Case–Control Study. Biol. Trace Elem. Res..

[5] Mihailović M., Cvetkovic M., Ljubić A., Kosanović M., Nedeljković S., Jovanovic I., Pesut O., Cvetkovč M. (2000). Selenium and Malondialdehyde Content and Glutathione Peroxidase Activity in Maternal and Umbilical Cord Blood and Amniotic Fluid. Biol. Trace Elem. Res..

[6] Abdulah R., Noerjasin H., Septiani L., Mutakin, Defi I.R., Suradji E.W., Puspitasari I.M., Barliana M.I., Yamazaki C., Nakazawa M. (2013). Reduced serum selenium concentration in miscarriage incidence of Indonesian subjects. Biol. Trace Elem. Res..

[7] Lee B.J., Worland P.J., Davis J.N., Stadtman T.C., Hatfield D.L. (1989). Identification of a selenocysteyl-tRNA(Ser) in mammalian cells that recognizes the nonsense codon, UGA. J. Biol. Chem..

[8] Rayman M.P. (2000). The importance of selenium to human health. Lancet.

[9] Lu J., Holmgren A. (2009). Selenoproteins. J. Biol. Chem..

[10] Glass R.S., Singh W.P., Jung W., Veres Z., Scholz T., Stadtman T. (1993). Monoselenophosphate: Synthesis, characterization, and identity with the prokaryotic biological selenium donor, compound SePX. Biochemistry.

[11] Guimarães M.J., Peterson D., Vicari A., Cocks B.G., Copeland N.G., Gilbert D.J., Jenkins N.A., Ferrick D.A., Kastelein R.A., Bazan J.F. (1996). Identification of a novel selD homolog from Eukaryotes, Bacteria, and Archaea: Is there an autoregulatory mechanism in selenocysteine metabolism?. Proc. Natl. Acad. Sci. USA.

[12] Low S.C., Harney J.W., Berry M.J. (1995). Cloning and Functional Characterization of Human Selenophosphate Synthetase, an Essential Component of Selenoprotein Synthesis. J. Biol. Chem..

[13] Xu X.M., Carlson B.A., Irons R., Mix H., Zhong N., Gladyshev V.N., Hatfield D.L. (2007). Selenophosphate synthetase 2 is essential for selenoprotein biosynthesis. Biochem. J..

[14] Dickinson M.E., Flenniken A.M., Ji X., Teboul L., Wong M.D., White J.K., Meehan T.F., Weninger W.J., Westerberg H., Adissu H. (2016). High-throughput discovery of novel developmental phenotypes. Nature.

[15] Alsina B., Serras F., Baguñà J., Corominas M. (1998). patufet, the gene encoding the Drosophila melanogaster homologue of selenophosphate synthetase, is involved in imaginal disc morphogenesis. Mol. Genet. Genom..

[16] Serras F., Morey M., Alsina B., Baguna J., Corominas M. (2001). The Drosophila selenophosphate synthetase (selD) gene is required for development and cell proliferation. Biofactors.

[17] Morey M., Corominas M., Serras F. (2003). DIAP1 suppresses ROS-induced apoptosis caused by impairment of the selD/sps1 homolog in Drosophila. J. Cell Sci..

[18] Shim M.S., Kim J.Y., Jung H.K., Lee K.H., Xu X.-M., Carlson B.A., Kim K.W., Kim I.Y., Hatfield D.L., Lee B.J. (2009). Elevation of Glutamine Level by Selenophosphate Synthetase 1 Knockdown Induces Megamitochondrial Formation in Drosophila Cells. J. Biol. Chem..

[19] Tobe R., Carlson B.A., Huh J.H., Castro N.P., Xu X.-M., Tsuji P.A., Lee S.-G., Bang J., Na J.-W., Kong Y.-Y. (2016). Selenophosphate synthetase 1 is an essential protein with roles in regulation of redox homoeostasis in mammals. Biochem. J..

[20] Noda Y., Matsumoto H., Umaoka Y., Tatsumi K., Kishi J., Mori T. (1991). Involvement of superoxide radicals in the mouse two-cell block. Mol. Reprod. Dev..

[21] Guérin P., El Mouatassim S., Ménézo Y. (2001). Oxidative stress and protection against reactive oxygen species in the pre-implantation embryo and its surroundings. Hum. Reprod. Update.

[22] Rodríguez-González E., López-Bejar M., Mertens M.-J., Paramio M.-T. (2003). Effects on in vitro embryo development and intracellular glutathione content of the presence of thiol compounds during maturation of prepubertal goat oocytes. Mol. Reprod. Dev..

[23] Matsui M., Oshima M., Oshima H., Takaku K., Maruyama T., Yodoi J., Taketo M.M. (1996). Early Embryonic Lethality Caused by Targeted Disruption of the Mouse Thioredoxin Gene. Dev. Biol..

[24] Nonn L., Williams R.R., Erickson R.P., Powis G. (2003). The Absence of Mitochondrial Thioredoxin 2 Causes Massive Apoptosis, Exencephaly, and Early Embryonic Lethality in Homozygous Mice. Mol. Cell. Biol..

[25] Xin M., Davis C.A., Molkentin J., Lien C.-L., Duncan S., Richardson J.A., Olson E.N. (2006). A threshold of GATA4 and GATA6 expression is required for cardiovascular development. Proc. Natl. Acad. Sci. USA.

[26] Huang S., Shen Q., Mao W.-G., Li A.-P., Ye J., Liu Q.-Z., Zou C.-P., Zhou J.-W. (2006). JWA, a novel signaling molecule, involved in the induction of differentiation of human myeloid leukemia cells. Biochem. Biophys. Res. Commun..

[27] Tao G., Levay A., Gridley T., Lincoln J. (2011). Mmp15 is a direct target of Snai1 during endothelial to mesenchymal transformation and endocardial cushion development. Dev. Biol..

[28] Li H., Zhao L., Lau Y.S., Zhang C., Han R. (2021). Genome-wide CRISPR screen identifies LGALS2 as an oxidative stress-responsive gene with an inhibitory function on colon tumor growth. Oncogene.

[29] Wood H., Episkopou V. (1999). Comparative expression of the mouse Sox1, Sox2 and Sox3 genes from pre-gastrulation to early somite stages. Mech. Dev..

[30] Ip C.K., Fossat N., Jones V., Lamonerie T., Tam P.P.L. (2014). Head formation: OTX2 regulates Dkk1 and Lhx1 activity in the anterior mesendoderm. Development.

[31] Burtscher I., Lickert H. (2009). Foxa2 regulates polarity and epithelialization in the endoderm germ layer of the mouse embryo. Development.

[32] Arnold S.J., Hofmann U.K., Bikoff E.K., Robertson E.J. (2008). Pivotal roles for eomesodermin during axis formation, epithelium-to-mesenchyme transition and endoderm specification in the mouse. Development.

[33] Nowotschin S., Costello I., Piliszek A., Kwon G.S., Mao C.-A., Klein W.H., Robertson E.J., Hadjantonakis A.-K. (2013). The T-box transcription factor Eomesodermin is essential for AVE induction in the mouse embryo. Genes Dev..

[34] Linderholm A.L., Onitsuka J., Xu C., Chiu M., Lee W.-M., Harper R.W. (2010). All-trans retinoic acid mediates DUOX2 expression and function in respiratory tract epithelium. Am. J. Physiol. Cell. Mol. Physiol..

[35] Kam R.K.T., Deng Y., Chen Y., Zhao H. (2012). Retinoic acid synthesis and functions in early embryonic development. Cell Biosci..

[36] Simeonov K.P., Uppal H. (2014). Direct Reprogramming of Human Fibroblasts to Hepatocyte-Like Cells by Synthetic Modified mRNAs. PLoS ONE.

[37] Kuckenberg P., Kubaczka C., Schorle H. (2012). The role of transcription factor Tcfap2c/TFAP2C in trophectoderm development. Reprod. Biomed. Online.

[38] Teichweyde N., Kasperidus L., Carotta S., Kouskoff V., Lacaud G., Horn P.A., Heinrichs S., Klump H. (2018). HOXB4 Promotes Hemogenic Endothelium Formation without Perturbing Endothelial Cell Development. Stem Cell Rep..

[39] Steinhart Z., Angers S. (2018). Wnt signaling in development and tissue homeostasis. Development.

[40] Subramanian A., Tamayo P., Mootha V.K., Mukherjee S., Ebert B.L., Gillette M.A., Paulovich A., Pomeroy S.L., Golub T.R., Lander E.S. (2005). Gene set enrichment analysis: A knowledge-based approach for interpreting genome-wide expression profiles. Proc. Natl. Acad. Sci. USA.

[41] Aman A., Piotrowski T. (2010). Cell migration during morphogenesis. Dev. Biol..

[42] Berge D.T., Koole W., Fuerer C., Fish M., Eroglu E., Nusse R. (2008). Wnt Signaling Mediates Self-Organization and Axis Formation in Embryoid Bodies. Cell Stem Cell.

[43] Nusse R., Lim X. The Wnt. http://web.stanford.edu/group/nusselab/cgi-bin/wnt/.

[44] Martinez-Alarcon O., García-Lopez G., Mora J.R.G., Molina-Hernandez A., Diaz-Martinez N.E., Portillo W., Diaz N.F. (2021). Prolactin from Pluripotency to Central Nervous System Development. Neuroendocrinology.

[45] Hartnett L., Glynn C., Nolan C.M., Grealy M., Byrnes L. (2010). Insulin-like growth factor-2 regulates early neural and cardiovascular system development in zebrafish embryos. Int. J. Dev. Biol..

[46] Shrivastav S.V., Bhardwaj A., Pathak K.A., Shrivastav A. (2020). Insulin-Like Growth Factor Binding Protein-3 (IGFBP-3): Unraveling the Role in Mediating IGF-Independent Effects within the Cell. Front. Cell Dev. Biol..

[47] Carbon S., Ireland A., Mungall C.J., Shu S., Marshall B., Lewis S., Ami G.O.H., Web Presence Working Group (2009). AmiGO: Online access to ontology and annotation data. Bioinformatics.

[48] Frazee A.C., Pertea G., Jaffe A.E., Langmead B., Salzberg S.L., Leek J.T. (2015). Ballgown bridges the gap between transcriptome assembly and expression analysis. Nat. Biotechnol..

[49] Love M.I., Huber W., Anders S. (2014). Moderated estimation of fold change and dispersion for RNA-seq data with DESeq2. Genome Biol..

[50] Robinson M.D., McCarthy D.J., Smyth G.K. (2010). edgeR: A Bioconductor package for differential expression analysis of digital gene expression data. Bioinformatics.

[51] Oki S., Ohta T., Shioi G., Hatanaka H., Ogasawara O., Okuda Y., Kawaji H., Nakaki R., Sese J., Meno C. (2018). ChIP-Atlas: A data-mining suite powered by full integration of public ChIP-seq data. EMBO Rep..

[52] Reich M., Liefeld T., Gould J., Lerner J., Tamayo P., Mesirov J.P. (2006). GenePattern 2.0. Nat. Genet..

[53] Kim D., Paggi J.M., Park C., Bennett C., Salzberg S.L. (2019). Graph-based genome alignment and genotyping with HISAT2 and HISAT-genotype. Nat. Biotechnol..

[54] Zhou Y., Zhou B., Pache L., Chang M., Khodabakhshi A.H., Tanaseichuk O., Benner C., Chanda S.K. (2019). Metascape provides a biologist-oriented resource for the analysis of systems-level datasets. Nat. Commun..

[55] Pijuan-Sala B., Griffiths J.A., Guibentif C., Hiscock T.W., Jawaid W., Calero-Nieto F.J., Mulas C., Ibarra-Soria X., Tyser R.C.V., Ho D.L.L. (2019). A single-cell molecular map of mouse gastrulation and early organogenesis. Nature.

[56] Bult C.J., Blake J.A., Smith C.L., Kadin J.A., Richardson J.E., Mouse Genome Database G. (2019). Mouse Genome Database (MGD) 2019. Nucleic Acids Res..

[57] Liberzon A., Birger C., Thorvaldsdottir H., Ghandi M., Mesirov J.P., Tamayo P. (2015). The Molecular Signatures Database (MSigDB) hallmark gene set collection. Cell Syst..

[58] Mi H., Ebert D., Muruganujan A., Mills C., Albou L.P., Mushayamaha T., Thomas P.D. (2021). PANTHER version 16: A revised family classification, tree-based classification tool, enhancer regions and extensive API. Nucleic Acids Res..

[59] Pertea M., Pertea G.M., Antonescu C.M., Chang T.C., Mendell J.T., Salzberg S.L. (2015). StringTie enables improved reconstruction of a transcriptome from RNA-seq reads. Nat. Biotechnol..

[60] Page R.D. (1996). TreeView: An application to display phylogenetic trees on personal computers. Comput. Appl. Biosci..

[61] Han H., Cho J.W., Lee S., Yun A., Kim H., Bae D., Yang S., Kim C.Y., Lee M., Kim E. (2018). TRRUST v2: An expanded reference database of human and mouse transcriptional regulatory interactions. Nucleic Acids Res..

[62] Hsu C.-W., Wong L., Rasmussen T.L., Kalaga S., McElwee M.L., Keith L.C., Bohat R., Seavitt J.R., Beaudet A.L., Dickinson M.E. (2016). Three-dimensional microCT imaging of mouse development from early post-implantation to early postnatal stages. Dev. Biol..

