# Supplementary material for: Identification of Signaling Pathways for Early Embryonic Lethality and Developmental Retardation in Sephs1−/− Mice
Source: Int J Mol Sci. 2021 Oct 28;22(21):11647. doi: 10.3390/ijms222111647 (PMC8583877; doi:10.3390/ijms222111647)
Supplement: Supplementary file 1 [file ijms-22-11647-s001.zip › Supplementary Information only.pdf]

International Journal of
Molecular Sciences

MDPI

Supplementary Information

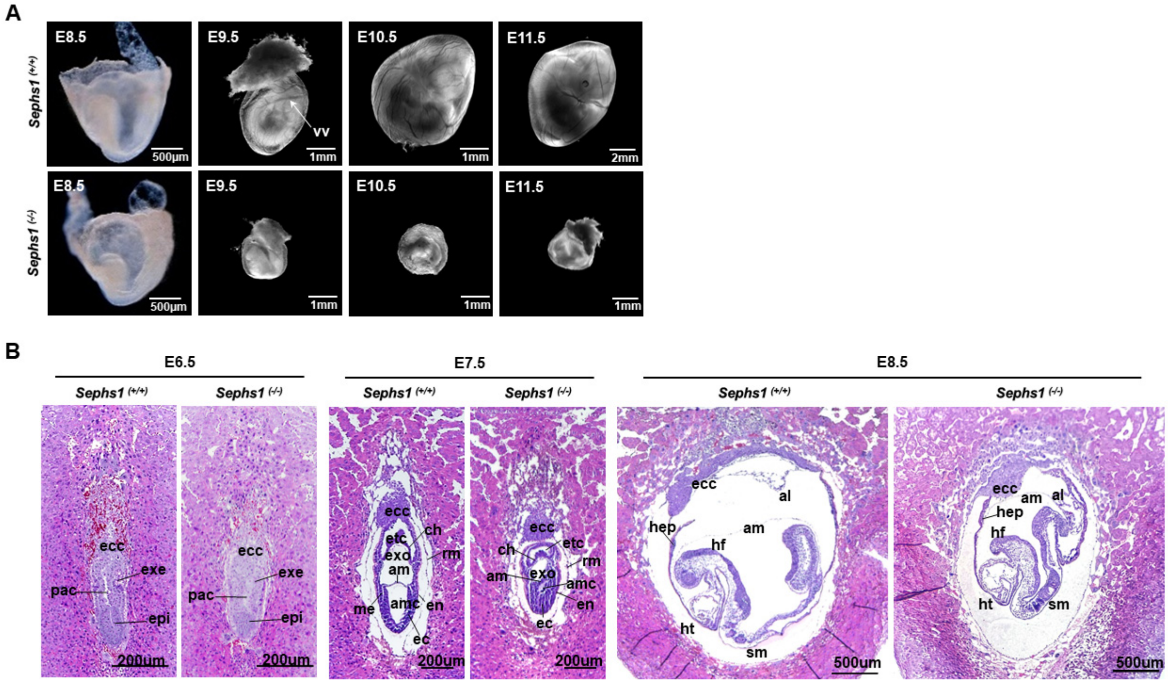

Figure S1. Morphology of *Sephs1*$^{-/-}$ embryos. **(A)** Embryos were photographed in the yolk sac immediately after dissection. **(B)** Paraffin-embedded section of *Sephs1*$^{-/-}$ embryo stained with Hematoxylin & eosin stain. exe, extraembryonic region; epi, epiblast; pac, pro-amniotic cavity; ecc, ectoplacental cone; am, amnion; etc, ectoplacental cavity; exo, exocoelomic cavity; amc, amiotic cavity; ec, ectoderm; en, endoderm; me, mesoderm; ch, chorion; hep, hemato-endothelial progenitor; hf, head fold; ht, heart; sm, somite; and al, allantois.

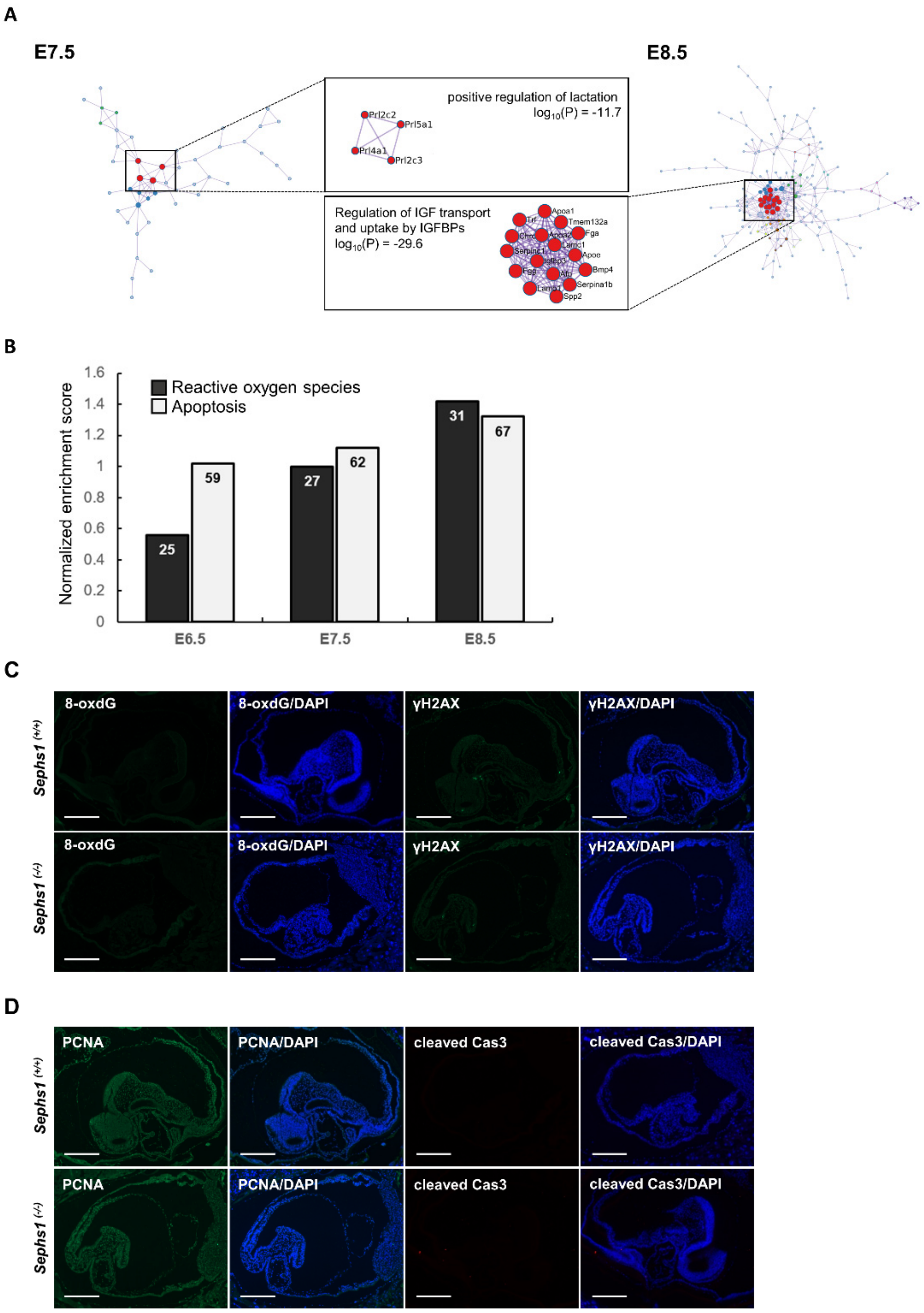

Figure S2. PPI analysis and GSEA on 'Reactive Oxygen Species' and 'Apoptosis' gene sets. (**A**) PPI analysis at E7.5 and E8.5. **(B)** Normalized enrichment score of GSEA with gene sets of 'Reactive Oxygen Species' and 'Apoptosis'. Each number in the bar graph indicates the number of genes in the LES. **(C)** Level of DNA damage induced by oxidative stress markers at E8.5. Scale bars represent 500 μm. **(D)** Level of proliferation marker and apoptosis marker at E8.5. Scale bars represent 500 μm.

Table S1. Top 25 up-regulated and top 25 down-regulated DEGs lists at E6.5, E7.5 and E8.5.

Top 25 downregulated at E6.5

Gene_symbol	Description	p-val	log2FC
Hba-a2	hemoglobin alpha, adult chain 2	9.81E-03	-8.78
Foxc2	forkhead box C2	6.35E-05	-4.41
Hsd11b1	hydroxysteroid 11-beta dehydrogenase 1	8.33E-04	-3.94
Cntnap2	contactin associated protein-like 2	9.56E-03	-3.71
Vat1l	vesicle amine transport protein 1 like	2.50E-04	-3.45
Amica1	adhesion molecule, interacts with CXADR antigen 1	3.47E-03	-3.23
Atp6v0d2	ATPase, H+ transporting, lysosomal V0 subunit D2	5.72E-03	-3.12
Rab39b	RAB39B, member RAS oncogene family	3.63E-04	-3.07
Ldhd	lactate dehydrogenase D	6.78E-05	-3.04
Ampd3	adenosine monophosphate deaminase 3	2.10E-03	-3.00
Mlph	melanophilin	6.84E-03	-2.95
Galnt16	UDP-N-acetyl-alpha-D-galactosamine:polypeptide N-acetyl	3.37E-05	-2.84
Fan1	FANCD2/FANCI-associated nuclease 1	9.44E-03	-2.83
Htra3	HtrA serine peptidase 3	6.39E-04	-2.83
Nlgn3	neuroligin 3	4.02E-03	-2.77
Gpnmb	glycoprotein (transmembrane) nmb	1.14E-03	-2.76
Sprr2g	small proline-rich protein 2G	4.11E-03	-2.75
Erv3	endogenous retroviral sequence 3	6.81E-03	-2.70
Acss1	acyl-CoA synthetase short-chain family member 1	5.04E-03	-2.65
Wnt3a	wingless-type MMTV integration site family, member 3A	8.36E-04	-2.65
Prap1	proline-rich acidic protein 1	6.91E-04	-2.54
Rbm24	RNA binding motif protein 24	2.54E-03	-2.50
Noto	notochord homeobox	8.77E-04	-2.43
Tmem213	transmembrane protein 213	1.99E-03	-2.42
Hephl1	hephaestin-like 1	4.89E-03	-2.36

Top 25 upregulated at E6.5

Gene_symbol	Description	p-val	log2FC
Prl4a1	prolactin family 4, subfamily a, member 1	4.61E+00	6.18
Prl3d2	prolactin family 3, subfamily d, member 1	4.34E+00	4.43
Prl5a1	prolactin family 5, subfamily a, member 1	3.67E+00	4.32
Prl7a1	prolactin family 7, subfamily a, member 1	3.73E+00	4.10
Nppb	natriuretic peptide type B	3.01E+00	3.87
Cts8	cathepsin 8	2.88E+00	3.61
Cts7	cathepsin 7	3.36E+00	3.53
Pfpl	pore forming protein-like	2.92E+00	3.18
Bmp8b	bone morphogenetic protein 8b	2.23E+00	3.09
AU018091	expressed sequence AU018091	2.45E+00	3.03
Entpd1	ectonucleoside triphosphate diphosphohydrolase 1	2.08E+00	3.02
Atp2c2	ATPase, Ca++ transporting, type 2C, member 2	1.65E+00	2.95
Mmp9	matrix metallopeptidase 9	2.69E+00	2.90
Adm	adrenomedullin	2.66E+00	2.84
Duoxa2	dual oxidase maturation factor 2	1.92E+00	2.80
Rhox1	reproductive homeobox 1	1.48E+00	2.68
Scin	scinderin	2.19E+00	2.56
Itgb3	integrin beta 3	1.82E+00	2.55
Gjb5	gap junction protein, beta 5	2.20E+00	2.54
Gjb3	gap junction protein, beta 3	2.18E+00	2.54
Gpx1	glutathione peroxidase 1	4.88E-01	2.49
Gm1673	predicted gene 1673	1.97E+00	2.47
Elf5	E74-like factor 5	2.25E+00	2.39
Tacstd2	tumor-associated calcium signal transducer 2	1.83E+00	2.36
Plau	plasminogen activator, urokinase	1.41E+00	2.32

Top 25 downregulated at E7.5

Gene_symbol	Description	p-val	log2FC
Stmn2	stathmin-like 2	2.18E-03	-6.76
Tmem176a	transmembrane protein 176A	2.67E-03	-5.50
Vat1l	vesicle amine transport protein 1 like	1.29E-04	-5.33
Tmem100	transmembrane protein 100	3.45E-03	-4.92
Tmem35	transmembrane protein 35	1.14E-04	-4.78
Them6	thioesterase superfamily member 6	2.72E-03	-4.63
Rtn4rl2	reticulon 4 receptor-like 2	4.59E-03	-4.41
Cntnap2	contactin associated protein-like 2	3.65E-03	-4.35
Rab39b	RAB39B, member RAS oncogene family	7.88E-04	-4.11
Maneal	mannosidase, endo-alpha-like	7.26E-04	-3.95
Tmem132a	transmembrane protein 132A	2.06E-03	-3.70
Mfap2	microfibrillar-associated protein 2	3.24E-03	-3.64
Adck2	aarF domain containing kinase 2	1.06E-03	-3.60
Gsta4	glutathione S-transferase, alpha 4	1.51E-03	-3.05
Mtfp1	mitochondrial fission process 1	8.83E-03	-3.02
Vash1	vasohibin 1	3.61E-03	-2.78
Ghdc	GH3 domain containing	7.18E-04	-2.74
Lrfn3	leucine rich repeat and fibronectin type III domain containin	6.81E-03	-2.71
Fkbp10	FK506 binding protein 10	8.86E-03	-2.68
Ppp1r1a	protein phosphatase 1, regulatory (inhibitor) subunit 1A	5.72E-03	-2.62
Vwa5a	von Willebrand factor A domain containing 5A	5.18E-03	-2.57
Sh3gl2	SH3-domain GRB2-like 2	1.51E-03	-2.55
Gng3	guanine nucleotide binding protein (G protein), gamma 3	1.55E-03	-2.52
Fut10	fucosyltransferase 10	9.09E-04	-2.45
Nlgn3	neuroligin 3	8.56E-03	-2.39

Top 25 upregulated at E7.5

Gene_symbol	Description	p-val	log2FC
Prl2c3	prolactin family 2, subfamily c, member 3	2.69E+00	5.92
Slc6a2	solute carrier family 6 (neurotransmitter transporter, noradr	2.12E+00	5.07
Prl4a1	prolactin family 4, subfamily a, member 1	3.36E+00	4.95
Ceacam9	carcinoembryonic antigen-related cell adhesion molecule 9	1.81E+00	4.65
Cts8	cathepsin 8	2.44E+00	4.65
Grem2	gremlin 2, DAN family BMP antagonist	1.75E+00	4.54
Igfbp1	insulin-like growth factor binding protein 1	3.13E+00	4.32
Thbd	thrombomodulin	2.61E+00	4.30
Mmp1a	matrix metallopeptidase 1a (interstitial collagenase)	2.52E+00	4.30
Prl7a1	prolactin family 7, subfamily a, member 1	3.52E+00	4.00
Psg22	pregnancy-specific glycoprotein 22	2.59E+00	3.95
Pfpl	pore forming protein-like	2.80E+00	3.91
Lama1	laminin, alpha 1	3.65E+00	3.86
Cts7	cathepsin 7	3.27E+00	3.82
Prl5a1	prolactin family 5, subfamily a, member 1	2.63E+00	3.72
Mmp9	matrix metallopeptidase 9	2.88E+00	3.70
Serpina1b	serine (or cysteine) preptidase inhibitor, clade A, member 1	1.98E+00	3.57
Hs3st1	heparan sulfate (glucosamine) 3-O-sulfotransferase 1	2.69E+00	3.47
Ceacam15	carcinoembryonic antigen-related cell adhesion molecule 1	2.70E+00	3.42
Nid1	nidogen 1	3.15E+00	3.34
Prl2c2	prolactin family 2, subfamily c, member 2	2.69E+00	3.24
Srgn	serglycin	2.99E+00	3.20
Adm	adrenomedullin	2.93E+00	3.14
Hbb-bh1	hemoglobin Z, beta-like embryonic chain	2.61E+00	2.95
Cdh5	cadherin 5	2.83E+00	2.85

Top 25 downregulated at E8.5

Gene_symbol	Description	p-val	log2FC
Tmem176b	transmembrane protein 176B	7.39E-03	-5.41
Vat1l	vesicle amine transport protein 1 like	3.16E-03	-5.29
Serpinf1	serine (or cysteine) peptidase inhibitor, clade F, member 1	3.05E-03	-5.13
Cntnap2	contactin associated protein-like 2	5.18E-03	-5.00
Fxyd3	FXYD domain-containing ion transport regulator 3	7.04E-03	-4.69
Ghdc	GH3 domain containing	5.08E-03	-4.31
Tmem176a	transmembrane protein 176A	6.16E-03	-4.14
Slc6a2	solute carrier family 6 (neurotransmitter transporter, noradr	1.76E-06	-3.92
Galnt16	UDP-N-acetyl-alpha-D-galactosamine:polypeptide N-acetyl	4.50E-03	-3.87
Chrdl1	chordin-like 1	6.77E-04	-3.46
Prl4a1	prolactin family 4, subfamily a, member 1	2.93E-03	-3.38
Rtn4rl2	reticulon 4 receptor-like 2	9.25E-03	-3.23
Hsd3b6	hydroxy-delta-5-steroid dehydrogenase, 3 beta- and steroid	2.09E-03	-3.17
Mtfp1	mitochondrial fission process 1	1.12E-03	-3.09
Them6	thioesterase superfamily member 6	4.68E-03	-3.09
Loxl1	lysyl oxidase-like 1	1.02E-03	-2.99
Dnase1l3	deoxyribonuclease 1-like 3	8.09E-03	-2.97
Adck2	aarF domain containing kinase 2	2.04E-03	-2.95
Tmem132a	transmembrane protein 132A	1.41E-03	-2.84
Thbd	thrombomodulin	8.01E-04	-2.52
Lama1	laminin, alpha 1	9.63E-04	-2.47
Cyp17a1	cytochrome P450, family 17, subfamily a, polypeptide 1	9.33E-03	-2.45
Msx3	msh homeobox 3	2.75E-04	-2.44
Grem2	gremlin 2, DAN family BMP antagonist	8.25E-04	-2.43
Arl6ip5	ADP-ribosylation factor-like 6 interacting protein 5	1.80E-03	-2.43

Top 25 upregulated at E8.5

Gene_symbol	Description	p-val	log2FC
Prl2c4	prolactin family 2, subfamily c, member 4	4.70E-03	10.02
Orm2	orosomucoid 2	5.20E-03	5.49
Hephl1	hephaestin-like 1	1.46E-04	5.40
Erv3	endogenous retroviral sequence 3	2.03E-03	4.91
Klk7	kallikrein related-peptidase 7 (chymotryptic, stratum corneu	5.08E-03	4.76
Psca	prostate stem cell antigen	4.86E-04	4.49
Prl8a2	prolactin family 8, subfamily a, member 2	1.55E-04	4.21
Htra3	HtrA serine peptidase 3	5.11E-04	3.90
Gpnmb	glycoprotein (transmembrane) nmb	2.33E-03	3.87
Htra1	HtrA serine peptidase 1	6.06E-04	3.74
Gpihbp1	GPI-anchored HDL-binding protein 1	8.00E-04	3.72
Adamts5	a disintegrin-like and metallopeptidase (reprolysin type) wit	3.66E-04	3.37
Mfap5	microfibrillar associated protein 5	3.62E-03	3.25
Il1r2	interleukin 1 receptor, type II	3.86E-03	3.22
Ctla2a	cytotoxic T lymphocyte-associated protein 2 alpha	6.35E-03	2.86
A2m	alpha-2-macroglobulin	3.28E-04	2.76
Lipg	lipase, endothelial	6.87E-03	2.67
Psap	prosaposin	5.88E-04	2.64
Sprr2g	small proline-rich protein 2G	1.47E-03	2.55
Krt20	keratin 20	4.62E-03	2.32
Fabp4	fatty acid binding protein 4, adipocyte	6.20E-03	2.21
Rbp2	retinol binding protein 2, cellular	7.18E-03	2.03
S100a8	S100 calcium binding protein A8 (calgranulin A)	4.10E-03	2.01
Aoc1l2	amine oxidase copper containing 1-like 2	8.27E-03	1.96
Ambp	alpha 1 microglobulin/bikunin	4.94E-03	1.96

Table S2. Canonical pathways obtained from IPA

Embryonic Day	Name	$log_{10}(p)$*	Z score	LXR/RXR/FXR Gene list
E6.5	LXR/RXR Activation	-8.93	2.27	APOA1, APOA2, APOA4, APOC2, APOM, C3, FGA, IL1R1, KNG1, LPL, LY96, MMP9, NFKB1, NOS2, PLTP, RBP4, S100A8, SERPINF1, SERPINF2, TF, TTR
	Coagulation System	-6.75	1.90	A2M, FGA, FGB, FGG, KNG1, PLAU, PLAUR, SERPINE1, SERPINF2, THBD
	Regulation Of The Epithelial Mesenchymal Transition In Development Pathway	-6.62	-2.84	APH1B, FZD3, GLI1, JAG1, NFKB1, S100A4, SNAI2, TWIST1, WNT2, WNT2B, WNT3A, WNT5A, WNT6, WNT7B, WNT8A
E7.5	Coagulation System	-11.90	1.29	F2, F3, FGA, FGB, FGG, PLAT, PLAU, PLAUR, PLG, PROS1, SERPINA1, SERPINC1, SERPINF2, TFPI, THBD
	LXR/RXR Activation	-7.86	2.63	ABCA1, AMBP, APOA1, APOA2, APOA4, APOB, APOC2, FGA, GC, IL1R2, LCAT, LPL, LYZ, MMP9, NGFR, PLTP, RBP4, SERPINA1, SERPINF2, TF, TTR
	LPS/IL-1 Mediated Inhibition of RXR Function	-5.59	-1.16	ABCA1, ABCC2, ABCC4, ACOX1, APOC2, CHST11, CHST3, Cyp2d26, FABP1, FABP2, FABP3, FMO1, FMO4, GSTM2, Gstm3, GSTM4, GSTM5, HS3ST1, IL1R2, LIPC, MAOB, MAP2K7, MYD88, NGFR, PLTP, PPARGC1B, SULT1E1
E8.5	Coagulation System	-7.20	-1.00	A2M, F3, F5, FGA, FGG, KNG1, SERPINA1, SERPINF2, THBD
	LXR/RXR Activation	-6.87	1.29	AMBP, APOA2, APOA4, FGA, IL1R2, IL33, KNG1, LBP, RXRG, S100A8, SERPINA1, SERPINF1, SERPINF2, TF, TNFRSF1A
	Acute Phase Response Signaling	-5.72	1.60	ADAMTS5, CEBPB, CTNNA2, DCN, DDIT4, DDR2, FGF18, FZD9, GLI1, GREM1, HTRA1, IL1R2, ITGA2B, PTH1R, S100A8, SPHK1, Tcf7, TNFRSF1A, WNT8A

* Pathways with log10(p)<-5 were selected.

Table S3. List of genes in LES obtained from GSEA on 'Reactive Oxygen Species'.

Total		E6.5		E7.5		E8.5	
Gene symbol	RM score	Gene symbol	RM score	Gene symbol	RM score	Gene symbol	RM score
GLRX	1.221	PRDX6	1.044	PRDX6	1.727	GLRX	1.515
PRDX6	0.665	GLRX	0.97	PRDX2	1.264	NDUFB4	0.595
PRDX2	0.598	PRDX2	0.835	MGST1	1.251	HMOX2	0.518
NDUFS2	0.294	GPX3	0.684	GLRX	1.241	PTPA	0.307
NDUFB4	0.278	HHEX	0.543	NDUFB4	0.822	SOD2	0.266
HHEX	-0.154	NDUFB4	0.314	FES	0.81	GPX4	-0.261
MSRA	-0.16	SCAF4	0.283	MBP	0.627	GCLC	-0.275
SELENOS	-0.168	EGLN2	-0.363	NDUFS2	0.52	STK25	-0.277
LAMTOR5	-0.178	ATOX1	-0.363	ERCC2	0.4	LAMTOR5	-0.28
ATOX1	-0.196	PRDX1	-0.399	ABCC1	0.384	GCLM	-0.315
SCAF4	-0.224	GSR	-0.426	GSR	-0.3	MBP	-0.319
PRDX1	-0.231	PTPA	-0.457	SOD1	-0.39	MSRA	-0.329
SRXN1	-0.248	GPX4	-0.46	SCAF4	-0.489	FTL	-0.341
GPX4	-0.289	LAMTOR5	-0.478	TXN	-0.544	NDUFA6	-0.411
PRNP	-0.32	CAT	-0.492	CDKN2D	-0.592	SBNO2	-0.422
TXN	-0.34	PDLIM1	-0.61	PFKP	-0.619	GSR	-0.427
GSR	-0.365	CDKN2D	-0.707	LSP1	-0.684	CAT	-0.435
CAT	-0.478	SOD1	-0.72	TXNRD1	-0.726	ATOX1	-0.464
GCLM	-0.498	FTL	-0.843	GCLM	-0.777	SCAF4	-0.478
PFKP	-0.542	PFKP	-0.895	TXNRD2	-0.896	FES	-0.578
PDLIM1	-0.577	TXNRD1	-0.916	FTL	-0.904	CDKN2D	-0.592
TXNRD1	-0.597	GCLM	-0.949	CAT	-0.986	PRDX1	-0.633
SOD1	-0.602	TXNRD2	-1.28	PDLIM1	-1.179	SOD1	-0.696
CDKN2D	-0.635	JUNB	-1.657	NQO1	-1.216	TXN	-0.727
FTL	-0.707	NQO1	-2.043	GPX3	-1.416	HHEX	-0.891
JUNB	-0.744			JUNB	-2.065	TXNRD2	-0.905
TXNRD2	-0.914			SRXN1	-2.342	JUNB	-1.008
NQO1	-1.303					SRXN1	-1.281
						GPX3	-1.3
						PRNP	-2.155
						NQO1	-2.641

* RM score; rank metric score.

Table S4. List of genes in LES obtained from GSEA on 'Apoptosis'.

Total		E6.5		E7.5		E8.5	
Gene symbol	RM score	Gene symbol	RM score	Gene symbol	RM score	Gene symbol	RM score
MADD	1.425	MADD	2.905	MADD	3.048	LMNA	2.225
CCND1	1.117	CCND2	2.735	CD44	2.032	FEZ1	2.091
CASP8	1.057	CD44	2.344	BMF	1.587	EMP1	2.08
PPP2R5B	0.786	ATF3	1.744	CASP8	1.307	CD44	1.921
CD44	0.732	CASP8	1.468	CCND1	1.154	SATB1	1.883
CASP3	0.669	LEF1	1.353	PPP2R5B	1.126	CAV1	1.581
BMF	0.669	BMF	1.316	CDKN1B	1.028	MADD	1.537
CDKN1B	0.589	CDKN1B	1.257	CASP3	1.017	BMF	1.129
LEF1	0.549	F2R	1.245	BIK	0.969	CCND1	1.089
PAK1	0.459	SAT1	1.203	DIABLO	0.789	NEDD9	1.063
CASP2	0.41	LGALS3	1.195	IGFBP6	0.781	BGN	0.999
DIABLO	0.34	DAP	1.185	LEF1	0.765	PLAT	0.862
TIMP3	0.301	CASP3	1.129	PAK1	0.714	TIMP2	0.838
TIMP2	0.298	CCND1	1.116	RARA	0.711	PPP2R5B	0.785
CASP7	0.291	JUN	1.084	GADD45A	0.644	CASP8	0.782
FEZ1	0.278	PMAIP1	1.007	SATB1	0.628	CCND2	0.753
BCL2L1	0.265	NEDD9	0.938	CASP2	0.586	PDGFRB	0.737
NEDD9	0.256	TIMP2	0.937	PSEN2	0.468	TIMP3	0.716
CTNNB1	0.218	ISG20	0.926	TSPO	0.451	CDKN1B	0.542
BAX	-0.168	DCN	0.912	HMGB2	0.448	SLC20A1	0.531
IRF1	-0.172	ROCK1	0.861	LMNA	-0.436	DIABLO	0.506
BCAP31	-0.173	CASP2	0.844	IRF1	-0.442	CASP7	0.491
IGF2R	-0.18	GSR	-0.426	DFFA	-0.459	ERBB3	0.472
IFNGR1	-0.191	GCH1	-0.431	RHOB	-0.509	RHOB	0.463
GSN	-0.219	BGN	-0.446	IFITM3	-0.536	FDXR	0.384
ROCK1	-0.219	BAX	-0.45	SC5D	-0.609	RARA	0.376
CD38	-0.224	IFITM3	-0.453	BGN	-0.612	GPX1	-0.417
DNM1L	-0.228	GPX4	-0.46	DNM1L	-0.625	GSR	-0.427
PPT1	-0.276	RARA	-0.461	CASP6	-0.632	BNIP3L	-0.427
SPTAN1	-0.277	CDKN1A	-0.463	DNAJC3	-0.647	BMP2	-0.49
SC5D	-0.278	SLC20A1	-0.472	KRT18	-0.666	IGF2R	-0.494
CFLAR	-0.285	FDXR	-0.488	ISG20	-0.67	HMGB2	-0.503
GPX4	-0.289	IGF2R	-0.545	CFLAR	-0.671	IFNGR1	-0.514
TNFRSF12A	-0.293	PLAT	-0.55	BMP2	-0.684	SMAD7	-0.543
ERBB3	-0.305	IGFBP6	-0.592	SQSTM1	-0.761	SQSTM1	-0.552
IFITM3	-0.332	SATB1	-0.611	RETSAT	-0.831	ROCK1	-0.56
HSPB1	-0.351	CDC25B	-0.663	TIMP1	-0.877	CYLD	-0.645
IER3	-0.352	IRF1	-0.688	ROCK1	-0.893	SOD1	-0.696
CDK2	-0.363	MGMT	-0.709	CD38	-0.94	KRT18	-0.707
GSR	-0.365	SOD1	-0.72	TGFBR3	-0.94	CFLAR	-0.719
RETSAT	-0.375	RETSAT	-0.764	BTG2	-1.001	CLU	-0.729
BCL2L2	-0.393	BIK	-0.891	DAP	-1.059	CASP6	-0.735
CASP6	-0.409	HSPB1	-0.921	PDCD4	-1.066	BAX	-0.749
ANKH	-0.427	SQSTM1	-0.923	IGF2R	-1.207	AIFM3	-0.762
TIMP1	-0.444	EMP1	-0.947	CLU	-1.22	BCL2L2	-0.793
GNA15	-0.444	TIMP1	-0.988	SAT1	-1.281	GADD45A	-0.834
BTG2	-0.521	PLCB2	-1.183	SMAD7	-1.284	GADD45B	-0.845
GADD45B	-0.548	GSTM1	-1.214	GPX3	-1.416	TSPO	-0.966
CLU	-0.554	CLU	-1.22	NEDD9	-1.448	PDCD4	-1.077
PLCB2	-0.595	ERBB3	-1.263	GADD45B	-1.538	IRF1	-1.114
SOD1	-0.602	TNFRSF12A	-1.302	EMP1	-1.546	TGFB2	-1.158
HMOX1	-0.608	KRT18	-1.386	ATF3	-1.607	SAT1	-1.183
H1-0	-0.613	F2	-1.447	HMOX1	-1.646	LGALS3	-1.294
CDKN1A	-0.614	GADD45B	-1.643	HSPB1	-1.74	GPX3	-1.3
SQSTM1	-0.757	TAP1	-1.656	IER3	-1.798	MGMT	-1.31
SMAD7	-0.766	SMAD7	-1.773	GSN	-1.906	IFITM3	-1.375
MGMT	-0.77	HMOX1	-2.45	PLAT	-1.921	F2	-1.403
PDCD4	-0.798	GNA15	-2.72	ANXA1	-2.257	BTG2	-1.432
KRT18	-0.867	GPX1	-3.487	LGALS3	-2.371	IER3	-1.472
GPX1	-1.054			F2	-2.447	CDKN1A	-1.545
GSTM1	-1.09			GSTM1	-2.531	GSN	-1.557
F2	-1.102			GPX1	-3.228	CDK2	-1.599
TAP1	-1.153					GSTM1	-1.677
						ANXA1	-1.799
						PMAIP1	-2.077
						ISG20	-2.499
						DCN	-3.063

* RM score; rank metric score.

Video S1: 3D reconstruction of wild-type embryo at E8.5

Video S2: 3D reconstruction of *Sephs1*$^{-/-}$ embryo at E8.5

Video S3: 3D reconstruction of wild-type embryo at E9.5

Video S4: 3D reconstruction of *Sephs1*$^{-/-}$ embryo at E9.5
