# Supplementary figures and images for: Identification of Signaling Pathways for Early Embryonic Lethality and Developmental Retardation in Sephs1−/− Mice
Source: Int J Mol Sci. 2021 Oct 28;22(21):11647. doi: 10.3390/ijms222111647 (PMC8583877; doi:10.3390/ijms222111647)

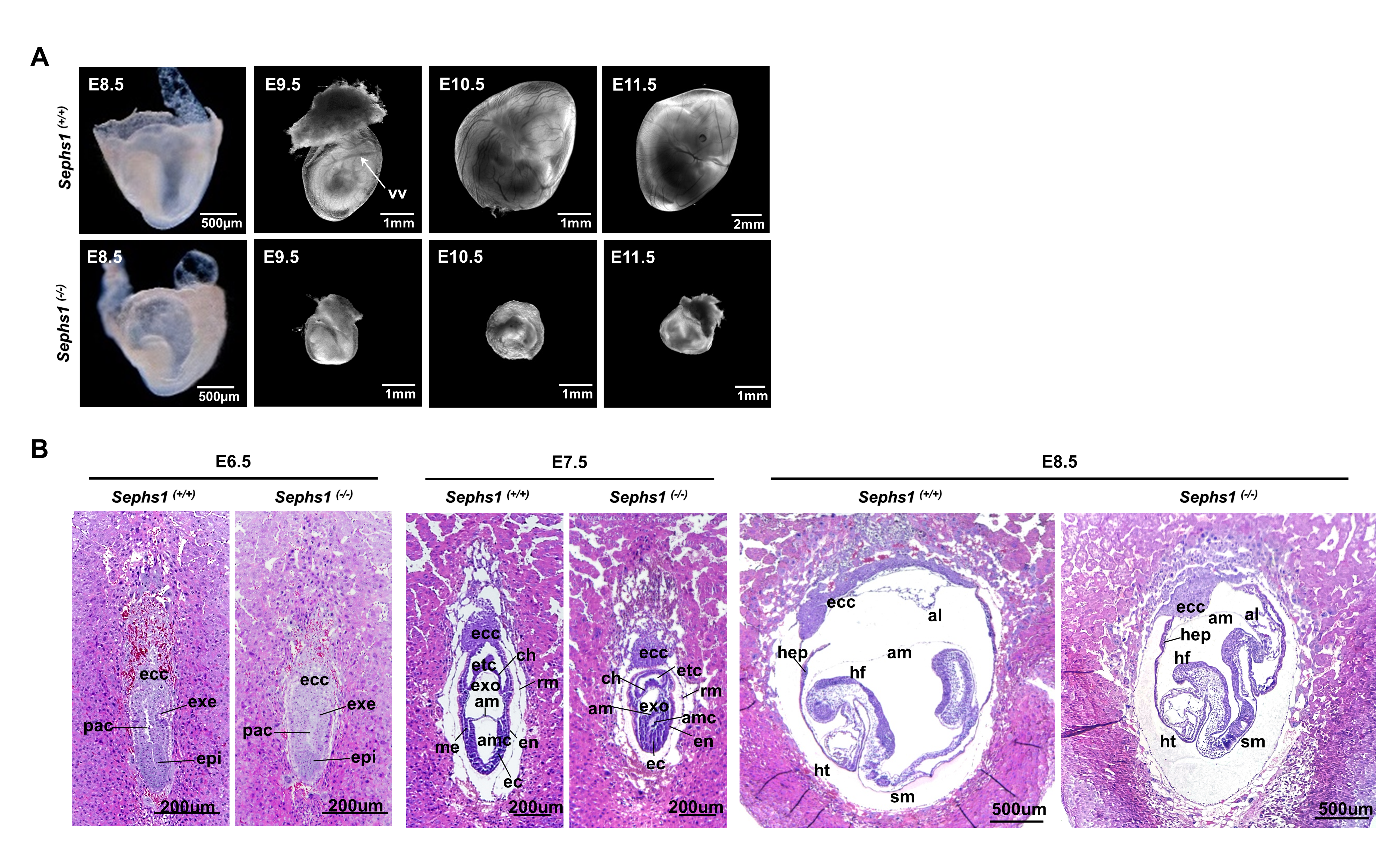

Supplement: Supplementary file 1 [file ijms-22-11647-s001.zip › Figure S1.bmp]

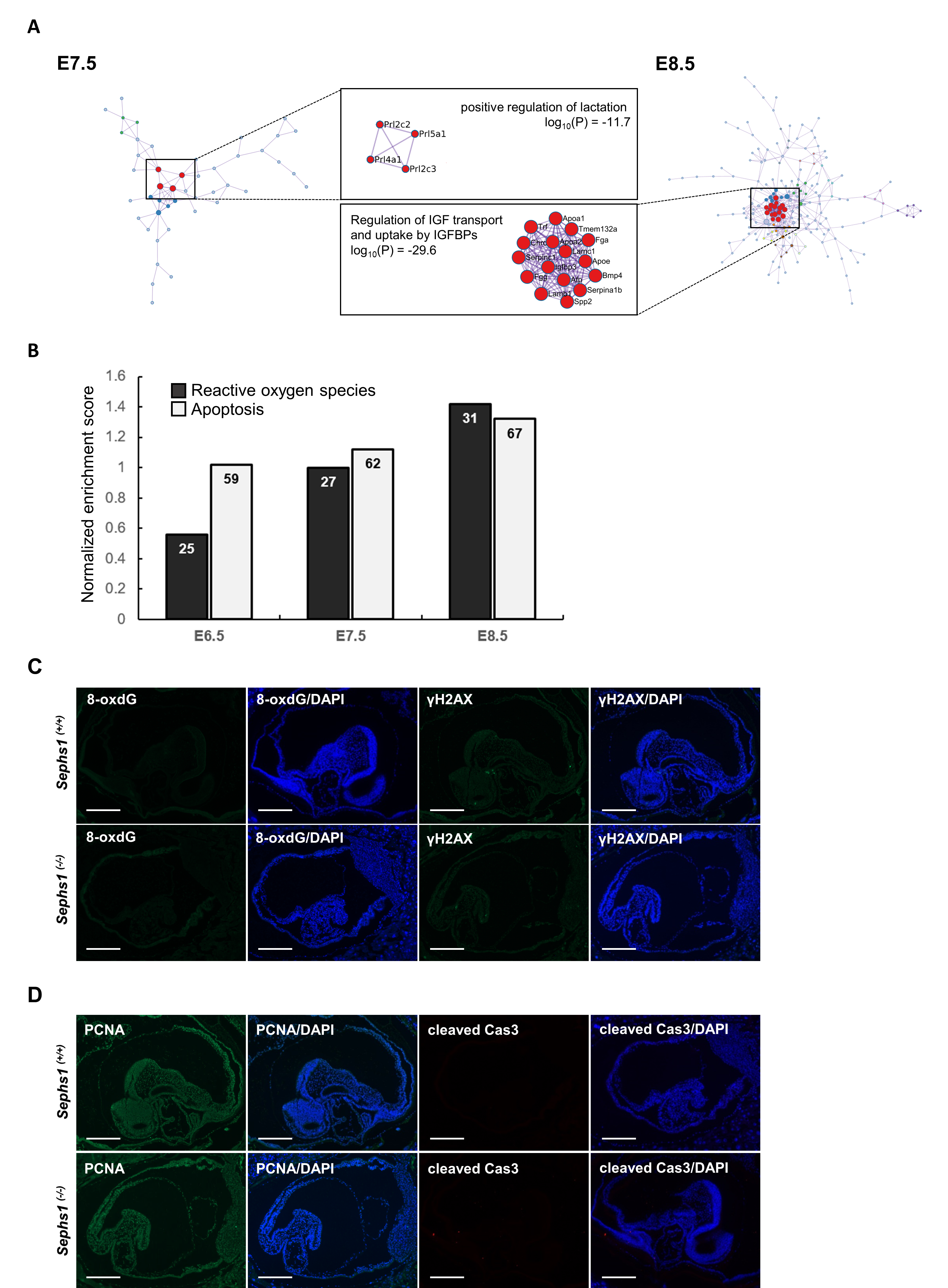

Supplement: Supplementary file 1 [file ijms-22-11647-s001.zip › Figure S2.bmp]

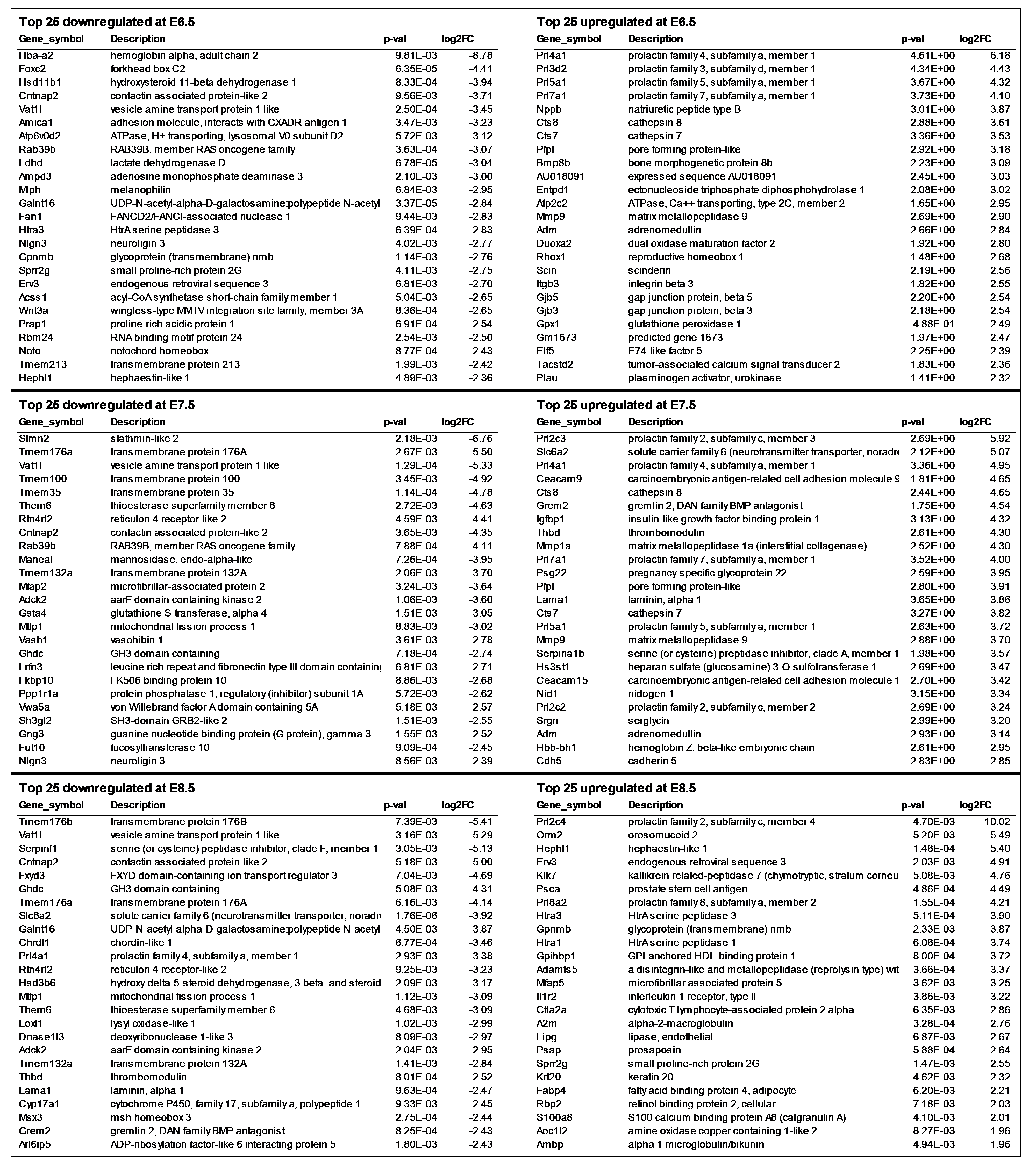

Supplement: Supplementary file 1 [file ijms-22-11647-s001.zip › Table S1.bmp]

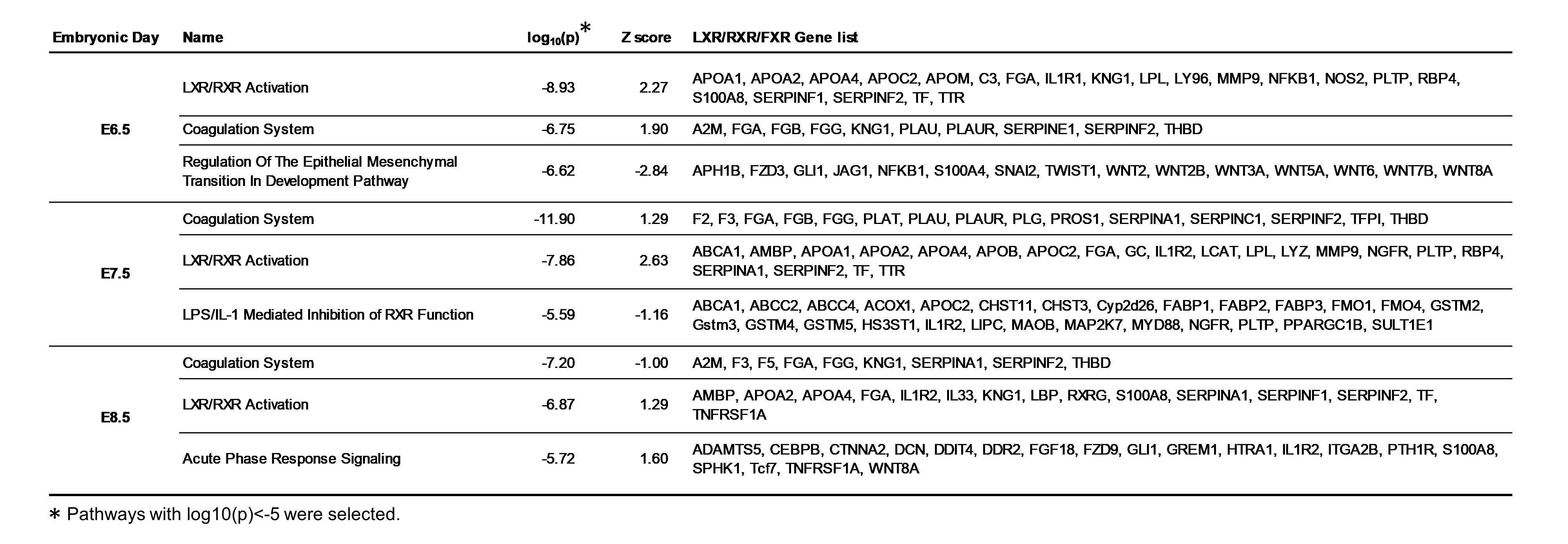

Supplement: Supplementary file 1 [file ijms-22-11647-s001.zip › Table S2.bmp]

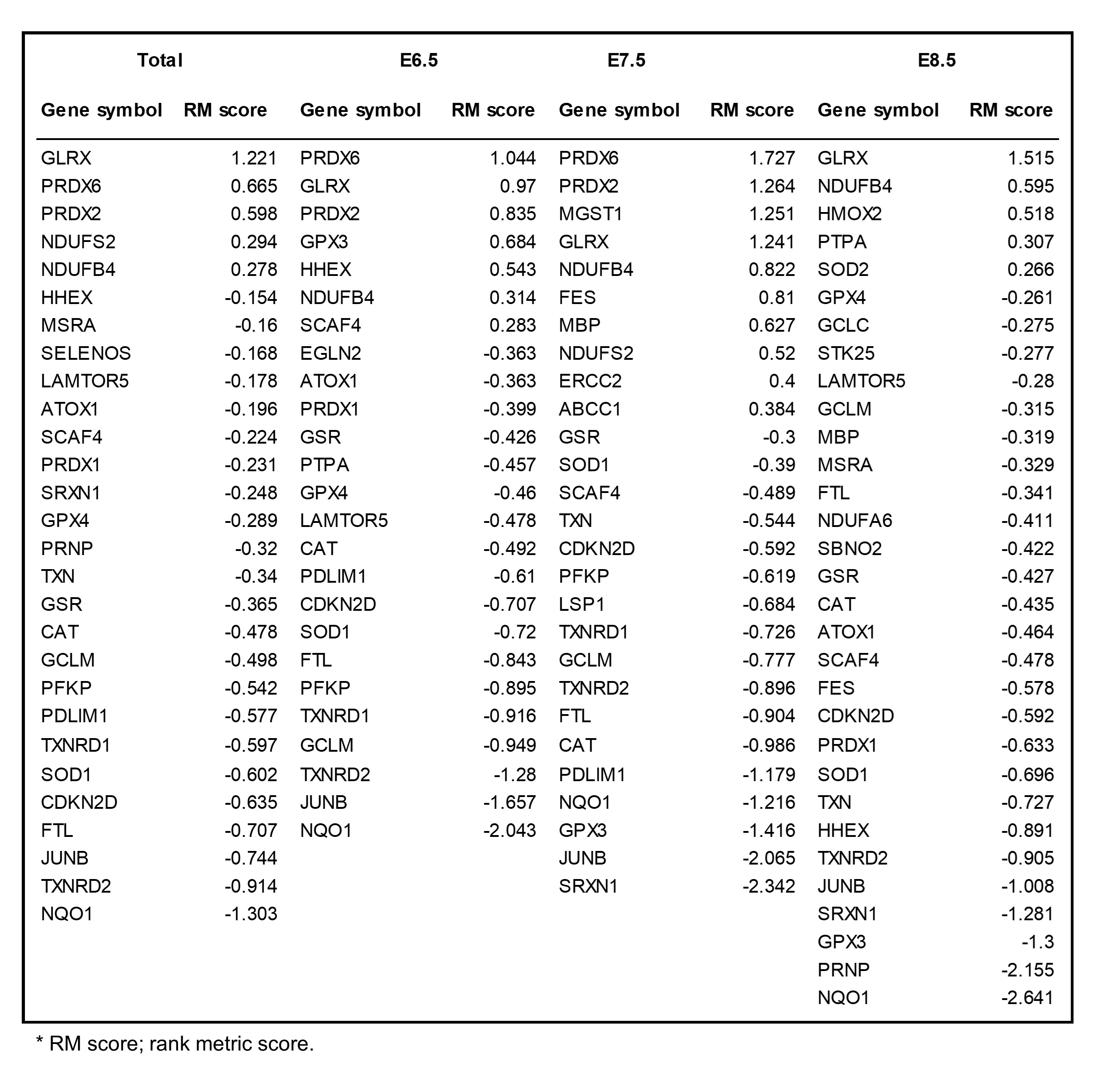

Supplement: Supplementary file 1 [file ijms-22-11647-s001.zip › Table S3.bmp]

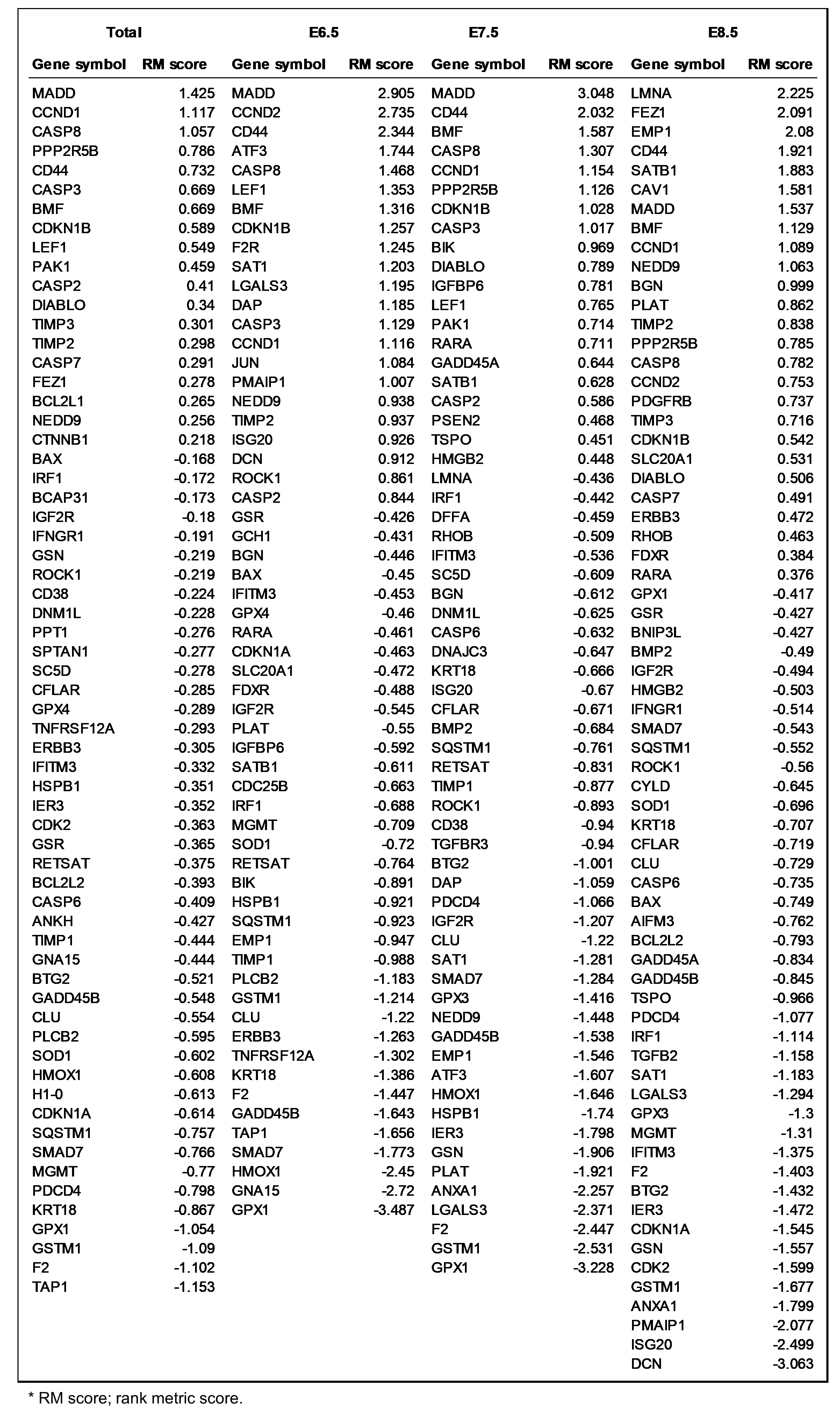

Supplement: Supplementary file 1 [file ijms-22-11647-s001.zip › Table S4.bmp]
